# Multicopy Suppressor Analysis of Strains Lacking Cytoplasmic Peptidyl-Prolyl *cis/trans* Isomerases Identifies Three New PPIase Activities in *Escherichia coli* That Includes the DksA Transcription Factor

**DOI:** 10.3390/ijms21165843

**Published:** 2020-08-14

**Authors:** Pawel Wojtkiewicz, Daria Biernacka, Patrycja Gorzelak, Anna Stupak, Gracjana Klein, Satish Raina

**Affiliations:** Unit of Bacterial Genetics, Gdansk University of Technology, Narutowicza 11/12, 80-233 Gdansk, Poland; pawwojtk1@student.pg.edu.pl (P.W.); darbiern@student.pg.edu.pl (D.B.); patrycja.gorzelak@gmail.com (P.G.); anna.stupak@pg.edu.pl (A.S.)

**Keywords:** prolyl isomerase, protein folding, RNA polymerase, DksA, heat shock proteins, DnaK/J, GroL/S, RpoE sigma factor, RNase H, LepA

## Abstract

Consistent with a role in catalyzing rate-limiting step of protein folding, removal of genes encoding cytoplasmic protein folding catalysts belonging to the family of peptidyl-prolyl *cis/trans* isomerases (PPIs) in *Escherichia coli* confers conditional lethality. To address the molecular basis of the essentiality of PPIs, a multicopy suppressor approach revealed that overexpression of genes encoding chaperones (DnaK/J and GroL/S), transcriptional factors (DksA and SrrA), replication proteins Hda/DiaA, asparatokinase MetL, Cmk and acid resistance regulator (AriR) overcome some defects of Δ6*ppi* strains. Interestingly, viability of Δ6*ppi* bacteria requires the presence of transcriptional factors DksA, SrrA, Cmk or Hda. DksA, MetL and Cmk are for the first time shown to exhibit PPIase activity in chymotrypsin-coupled and RNase T1 refolding assays and their overexpression also restores growth of a Δ(*dnaK/J/tig*) strain, revealing their mechanism of suppression. Mutagenesis of DksA identified that D74, F82 and L84 amino acid residues are critical for its PPIase activity and their replacement abrogated multicopy suppression ability. Mutational studies revealed that DksA-mediated suppression of either Δ6*ppi* or Δ*dnaK/J* is abolished if GroL/S and RpoE are limiting, or in the absence of either major porin regulatory sensory kinase EnvZ or RNase H, transporter TatC or LepA GTPase or P_i_-signaling regulator PhoU.

## 1. Introduction

To be functionally active, all newly synthesized polypeptides must rapidly fold into their native three-dimensional structure. It is now established that, right from the synthesis of a polypeptide from its nascent chain stage until it achieves its native structure, its maturation is to a large extent dependent on several factors. In bacteria, e.g., *Escherichia coli*, as well as in other organisms, these factors are molecular chaperones and folding catalysts, whose requirements vary, depending on the individual functional or folding requirement of client protein(s). In the cytosol, despite the molecular crowding, productive folding in the cell is achieved via some common strategies. Partially folded protein segments are bound by molecular chaperones to prevent their aggregation/misfolding and critical slow folding steps are accelerated by folding catalysts such as peptidyl-prolyl *cis/trans* isomerases (PPIs) and thiol-disulfide oxidoreductases, belonging to the protein disulfide isomerase (PDI) family, to reduce the accumulation of aggregation-prone folding intermediates [1,2].

Most of the peptide bonds in proteins are present in the *trans* configuration, since *trans* isomers are energetically more favorable than *cis* ones. However, proline is one exception, since it is an *N*-alkylated amino acid that creates an imidic peptide bond [3]. For the peptide bonds preceding proline, steric hindrance is comparable between two isomers. They are nearly isoenergetic (about 0.5 kcal/mol of free energy difference) and hence both conformations are thermodynamically possible [4,5]. Globally, around 7% proline residues are present in the *cis* conformation in different proteins in all three domains of life [6]. However, nonnative form of prolyl bond in a polypeptide chain can retard the completion of folding 10^3^–10^6^-fold [7]. PPIs are the only enzymes known thus far that can stabilize the transition state (high-energy state with ω around 90°) separated from the ground state by a difference in a torsional angle [3,4,5,6,7]. Thus, there is a requirement for PPIs to accelerate reactions that are rate-limited by the prolyl bond isomerization. PPIs are ubiquitous enzymes present in all organisms, virtually in all cell compartments and three families are known: the cyclophilins [8], the FK506-binding proteins (FKBPs) [9] and the parvulins [10]. The *E*. *coli* cytoplasm contains six PPIs, which cover all three families. However, their in vivo physiological requirement and specific substrates that require the PPIase activity have not been fully addressed.

We recently described the construction of strains devoid of all PPIs and showed that strains lacking all six cytoplasmic PPIs exhibit the synthetic lethality at high and low temperatures in rich medium [1]. Such Δ6*ppi* bacteria elicit a high propensity to accumulate proteins in the aggregation state and their identity revealed defects in folding of proteins involved in several essential processes. These include transcriptional-related proteins such as RNA polymerase subunits, the termination factor Rho, enzymes involved in metabolic processes and protein synthesis and those related to oxidative stress and DNA repair system. Several proteins that were identified to aggregate in Δ6*ppi* bacteria were found to be common to known aggregation-prone proteins in strains lacking the DnaK/J chaperone system [1]. Interestingly, measurement of the PPIase activity of a strain lacking all ten PPIs revealed a residual PPIase activity, suggesting the presence of some previously unknown PPI(s) [1].

**Figure 1:**
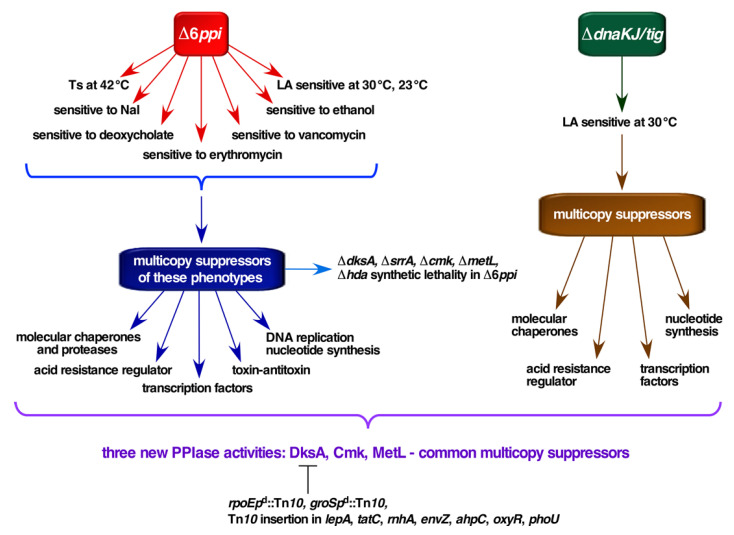
Schematic presentation of approach employed to identify factors that are limiting in Δ6*ppi* and Δ(*dnaK*/*J tig*) bacteria using multicopy suppressor approach. This identified several factors, whose overexpression can rescue their lethal phenotype. This identified transcription factor DksA, aspartokinase MetL and cytidylate kinase Cmk as common factors whose overproduction can restore growth and these factors also exhibit peptidyl *cis*/*trans* prolyl isomerase activity. Further, DksA-mediated suppression requires wild-type levels of GroL/S and RpoE, and it is abolished when either genome integrity is compromised in the absence of RNase H or in the absence of translational GTPase LepA or when cell envelope homeostasis is dysfunctional.

In this work, we used stringent high temperature and cold sensitive phenotypes of Δ6*ppi* bacteria to isolate multicopy suppressors that rescue some of their phenotypic defects (Figure 1). The results of such experiments reveal that overexpression of major chaperone systems (GroL/S and DnaK/J), transcriptional factors (DksA and SrrA), the cytidylate kinase Cmk and the aspartokinase MetL restored the growth of Δ6*ppi* strains under non-permissive growth conditions (Figure 1). Interestingly, DksA, Cmk and MetL were shown to exhibit the PPIase activity as well as suppress growth defects of a Δ(*dnaK/J tig*) strain. This PPIase activity was inhibited by the FK506 macrolide and mutations abrogating DksA’s PPIase activity were identified and shown to be defective in suppressing growth defects of Δ6*ppi* and Δ(*dnaK/J tig*) derivatives under non-permissive growth conditions. We further performed saturated mutagenesis in a *dksA*-overexpressing strain to isolate *trans*-acting mutations that block the multicopy suppression by DksA of Δ6*ppi* and Δ(*dnaK dnaJ*) strains, revealing a requirement for the intact GroL/S chaperone system, the RpoE sigma factor, the RNase H involved in genome integrity, the translation GTPase LepA and the TatC protein involved in protein translocation (Figure 1).

## 2. Results

### 2.1. Δ6ppi Mutant Bacteria Exhibit the Sensitivity towards Antibiotics, Membrane-Destabilizing Factors and the DNA-Damaging Agent Nalidixic Acid

**Figure 2:**
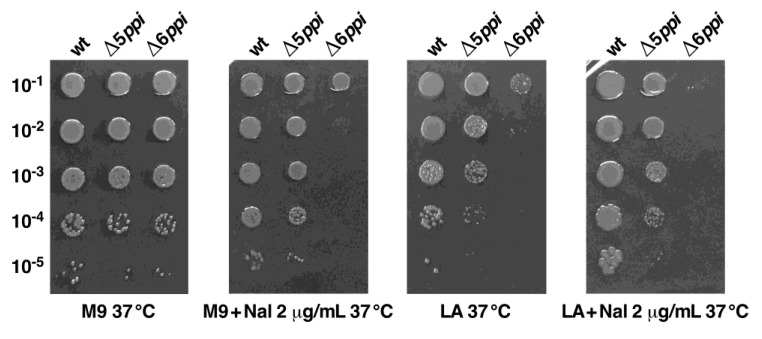
Δ6*ppi* bacteria exhibit the sensitivity to nalidixic acid (Nal) on either M9 minimal medium or Luria Agar (LA) medium. Exponentially grown cultures of the wild type and its isogenic Δ5*ppi* and Δ6*ppi* derivatives were adjusted to an optical density OD_600_ of 0.1 and serially spot diluted on M9 minimal medium and LA medium with or without supplementation with 2 μg/mL Nal. Plates were incubated at 37 °C for 24 h. Data presented are from one of the representative experiments.

It has been demonstrated that collectively the cytoplasmic PPIase activity is essential for the bacterial viability under optimal growth conditions [1]. To further understand the molecular basis of essentiality of cytoplasmic PPIs in *E*. *coli*, we examined growth properties and various defects exhibited by strains lacking six cytoplasmic PPIs. Besides cold- and temperature-sensitive phenotypes, Δ6*ppi* bacteria also reveal the sensitivity to mild challenge with ethanol (4%), membrane-destabilizing factors such as SDS (1%), deoxycholate (0.75%), vancomycin (60 μg/mL), erythromycin (12 μg/mL) and tetracycline (1.5 μg/mL) and aminoglycosides such as kanamycin (2 μg/mL) (Table 1). Quite strikingly, Δ6*ppi* bacteria exhibit hypersensitivity to the DNA-damaging agent nalidixic acid (Nal) (inability to grow above 2 μg/mL) on either M9 minimal medium or Luria Agar (LA) medium at 37 °C as compared to a Δ5*ppi* Δ(*ppiB fklB tig slyD ppiC*) strain (Figure 2). Nal is known to introduce both DNA-protein adducts and double-strand breaks by targeting the DNA gyrase and stabilizes gyrase-DNA cleavage complexes [11]. Such adducts can cause a barrier to DNA replication, transcription and chromosomal fragmentation [12]. The sensitivity of Δ6*ppi* bacteria to Nal suggests that PPIs might be required for folding of some components of the DNA replication/repair system and transcriptional apparatus. Overall, these results lead us to conclude that the PPIase activity is essential for the optimal growth and maintenance of the genome integrity.

### 2.2. Factors Limiting for the Viability of Δ6ppi Mutant Bacteria

**Figure 3:**
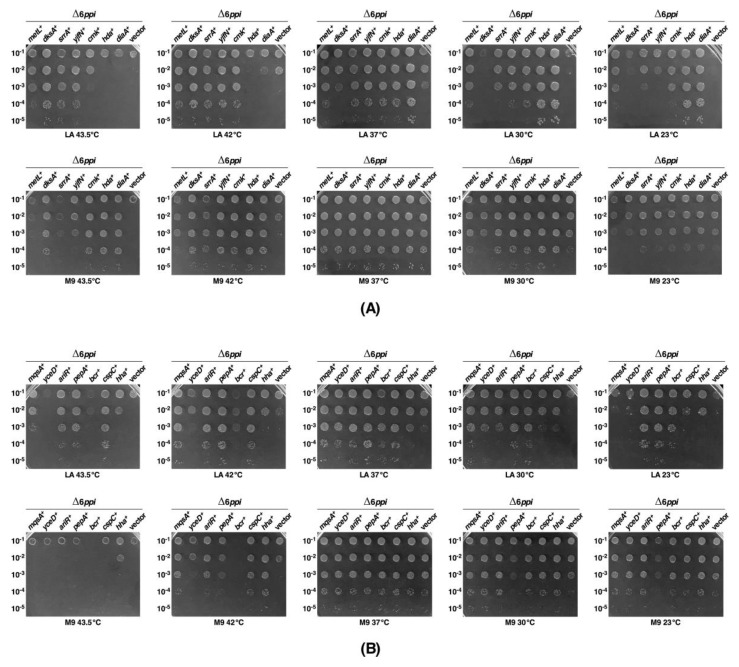
Identification of genes whose overexpression can restore the growth of Δ6*ppi* bacteria at different temperatures. Exponentially grown cultures of the wild type and its isogenic Δ6*ppi* derivatives transformed with either the vector pCA24N or the same vector expressing a specific gene under the control of IPTG-inducible promoter were grown at 37 °C in M9 minimal medium. Cultures were washed and adjusted to an optical density OD_600_ of 0.1 and serially spot diluted on M9 minimal medium and LA medium in the presence of 75 μM IPTG. (**A**,**B**) The growth of different strains expressing a specific gene with the indicated genotype, whose overexpression confers a varying degree of suppression depending upon temperature of incubation and growth medium.

We recently reported the construction of suppressor-free strains lacking all six cytoplasmic PPIs and showed that such bacteria are viable on M9 minimal medium at 37 °C, but unable to grow on rich medium at either low temperature (23 °C) or at temperatures above 37 °C [1]. To understand the molecular basis of growth defects of Δ6*ppi* mutant bacteria, a multicopy suppressor approach was undertaken in the Δ6*ppi* strain, using phenotypic defects such as the temperature-sensitive (Ts) phenotype and those with sensitivity towards detergents, ethanol, vancomycin and erythromycin. Two different multicopy libraries were employed. In one approach, plasmid DNA pools from the ASKA collection of single ORFs expressed from the tightly regulated IPTG-inducible P_T5_-*lac* promoter in the vector pCA24N [13] were introduced into the Δ6*ppi* strain SR18292. Transformants were selected for the restoration of growth on LA medium supplemented by 75 μM IPTG at different non-permissive growth conditions. The concentration of IPTG used for the controlled induction of expression of different genes was optimized as reported earlier [14,15]. In the second approach, a plasmid library in a p15A-based vector was constructed and upon transformation used to select for multicopy suppressors [16]. This library was constructed from the genomic DNA of Δ6*ppi* bacteria and introduced into the strain SR18292, selecting for the restoration of growth under non-permissive growth conditions. The construction of this library was necessitated to enrich the selection and allow cloning of those genes, which are organized as operons and whose products work together. The plasmid DNA was isolated from obtained suppressing clones and used to retransform the parental Δ6*ppi* strain to confirm the restoration of growth under non-permissive growth conditions. DNA sequence analysis identified several genes, whose overexpression allowed the growth on rich medium (at either high or low temperature) and suppressed the sensitivity towards different agents albeit to a different extent (Table 2). The extent of suppression was quantified by spot-dilution assays under different growth conditions in the presence of 75 μM IPTG as an inducer of gene expression, when plasmids from the ASKA collection were used (Figure 3). Some of the prominent genes encode well-characterized members of protein folding machinery (DnaK/J, GroL/S) (Table 2).

**Figure 4:**
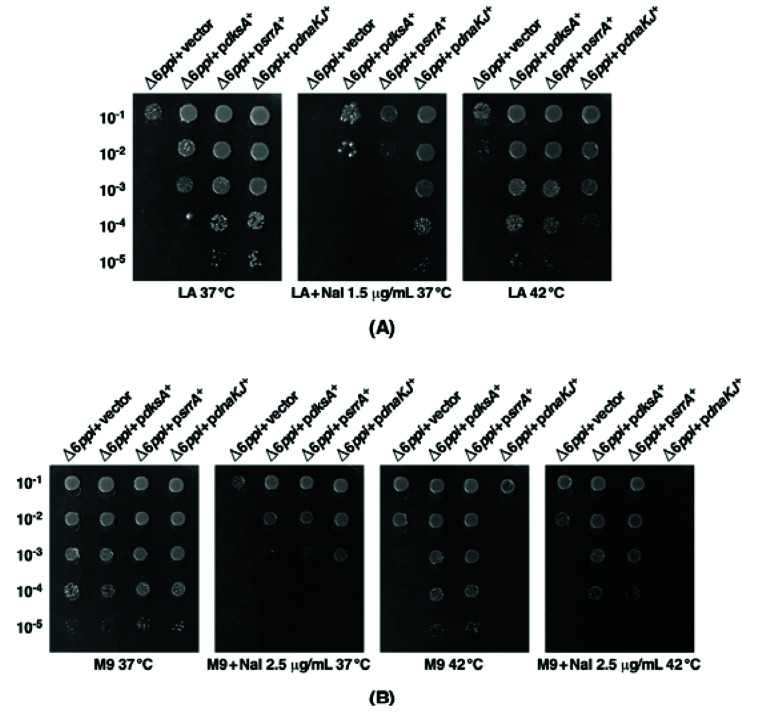
Overexpression of either the *dksA* gene or the *srrA* gene or the *dnaK/J* operon can rescue the sensitivity of Δ6*ppi* bacteria to nalidixic acid (Nal) to varying levels depending upon growth conditions. Exponentially grown cultures of the wild type and its isogenic Δ6*ppi* derivatives transformed with the vector pCA24N alone, the plasmid expressing either the *dksA* gene or the *srrA* gene or the *dnaK/J* operon were grown as described in the legend to Figure 3 and the bacterial growth measured on either LA medium (**A**) or on M9 medium (**B**) at indicated temperature. The concentration of Nal, when added to growth medium, is depicted. For the induction of gene expression, 75 μM IPTG was added.

Other multicopy suppressor-encoding genes include predicted or known transcription regulators [*dksA*, *srrA* (*yheO*), *ariR, ycjW*], an antitoxin *mqsA* and its potential interacting partner *hha*, while others are involved in DNA replication (*diaA*, *hda*), DNA/RNA synthesis (*cmk*), stress response and acid resistance (*ariR*, *gadW*, *yceD*, *cspC*), amino acid biosynthesis (*metL*), proteolytic turnover (*yjfN, pepA*), peptide transport (*bcr*, *mppA, ddpF*), peptidoglycan biosynthesis (*murI*) and nudix protein-encoding genes *nudE* and *nudG* (Table 2, Figure 3 and Supplementary Figure S1). At high temperature (42 °C), *dnaK*/*J* and *groS*/*L* operons, *dksA*, *srrA*, *yjfN*, *mqsA*, *ariR*, *pepA* and *cspC* were found to be major suppressors, whose overexpression restored the growth on rich medium with the efficiency of plating nearly to the wild-type level as determined by spot-dilution assays (Table 2, Figure 3 and Figure 4A). 

Among these, overexpression of *dksA*, *srrA, metL, cspC* and *yjfN* restored the wild type-like growth even up to 43.5 °C, conditions at which overexpression of either the *dnaK/J* or the *groS/L* genes confer only a partial suppression (Figure 3, Table 2 and Supplementary Table S1). Among various multicopy suppressors, overexpression of the *metL* gene encoding the aspartokinase II uniquely restored the wild type-like growth at low and high temperatures on LA medium (Figure 3). However, on M9 minimal medium at 43.5 °C, the *metL* overexpression does not provide the same relief as was observed on LA medium, although at 42 °C the restoration of growth of Δ6*ppi* bacteria was nearly to the wild-type level in either LA or M9 medium. The *srrA* gene is annotated as the *yheO* ORF with the unknown function. However, sequence examination of its coding sequence predicts it to be a DNA-binding transcriptional regulator and was designated SrrA (Stress Response Regulator A) based upon further characterization. It is worth noting that in multicopy the *dksA* gene does not exert any notable suppression at 37 °C or below, although it restores the growth at 43.5 °C with the efficiency of plating close to the wild type (Table 2 and Figure 3 and Figure 4). At low temperatures such as 23 or 30 °C, overexpression of *diaA*, *hda*, *ariR, pepA* and *ydgC* genes restored the wild type-like growth (Figure 3 and Supplementary Figure S1). Other multicopy suppressors that partially overcome the conditional lethality of Δ6*ppi* bacteria include products of heat shock genes such as Hsp33 and HchA, the cysteine detoxification protein YhaM and the RluB pseudouridine synthase (Table 2).

Regarding the suppression of sensitivity to erythromycin (15 μg/mL), ethanol (4.5%) and vancomycin (75 μg/mL), a multicopy plasmid library was used as described above, selecting for the restoration of growth of Δ6*ppi* bacteria on M9 minimal medium at 37 °C in the presence of either of these agents. DNA sequence analysis of suppressing clones after retransformation revealed that overexpression of *cmk*, *srrA*, *hda*, *diaA*, *bcr*, *murI* and *ddpF* nearly restored the wild type-like growth of Δ6*ppi* bacteria on M9 minimal medium at 37 °C in the presence of supplemented ethanol (4.5%) in the growth medium (Table 2). Interestingly, *metL*, *hda*, *srrA*, *cmk*, *yjfN* and *bcr* genes were again identified in the independent selection of multicopy suppressors for the restoration of growth in the presence of erythromycin antibiotic, in addition to *ariR*, *gadW*, *ycjW*, *mppA* and *murI* genes (Table 2). Concerning the selection of multicopy suppressors for the vancomycin sensitivity, *diaA*, *srrA*, *cmk*, *metL*, *yceD*, *mppA* and *murI* were cloned in this selection (Table 2). Interestingly, we also cloned the *eptB* gene, whose product is required for the modification of inner core of lipopolysaccharide by phosphoethanolamine [17], as a multicopy suppressor for the suppression of vancomycin sensitivity. Thus, several genes such as *srrA*, *hda*, *diaA*, *metL* and *cmk* were cloned in multiple approaches that overcome several defects of Δ6*ppi* bacteria and hence identify factors that are limiting in such PPIs-lacking bacteria.

### 2.3. Overexpression of Either the srrA Gene or the dksA Gene Suppresses the Sensitivity of Δ6ppi Bacteria to Nal

As Δ6*ppi* bacteria exhibit hypersensitivity to Nal, we tested if any of the major multicopy suppressors can also restore the growth on medium supplemented with Nal. Towards this goal, we mainly tested the effect of overexpression of *dksA*, *srrA* and *dnaK/J,* since they are among the most prominent multicopy suppressors of growth defects of Δ6*ppi* bacteria (Figure 3 and Figure 4A and Table 2). At 37 °C, overexpression of either *dnaK*/*J* or *srrA* genes restored the growth of Δ6*ppi* bacteria on M9 medium supplemented with 2.5 μg/mL of Nal nearly to the wild-type level, thereby suppressing the Nal-sensitivity phenotype (Figure 4B). In LA medium, when supplemented with 1.5 μg/mL of Nal, only overexpression of *dnaK*/*J* was able to suppress the Nal sensitivity at 37 °C (Figure 4A). However, at 42 °C, overexpression of either the *dksA* or the *srrA* gene could rescue the growth on M9 medium supplemented with Nal (Figure 4B). These results thus allow us to conclude that the Nal sensitivity can be overcome by overexpression of either *dksA* or *srrA* genes and only partly by overexpressing *dnaK*/*J* genes up to a temperature of 37 °C.

### 2.4. The Essentiality of DksA, SrrA, MetL and Hda for the Viability of Δ6ppi Bacteria and Synthetic Growth Defects with Δcmk and ΔhchA

If indeed any of the products of genes encoding different multicopy suppressors identified in above studies are limiting for the growth of Δ6*ppi* bacteria was investigated by introducing deletion mutations of their cognate genes in the Δ6*ppi* strain. The results of such experiments reveal that *dksA*, *srrA*, *metL* and *hda* genes are indispensable for the growth of Δ6*ppi* bacteria such as SR18292 as their deletion combinations were lethal (Table 3). The deletion of either the *dksA* gene or the *srrA* gene or the *hda* gene or the *metL* gene could be introduced in Δ6*ppi* bacteria on LA medium at 37 °C only if the wild-type copy of the corresponding gene was present on the plasmid. However, it is worth noting that a deletion of the *metL* gene can be tolerated on M9 minimal medium (supplemented by 0.2% casamino acids), but not of either the *srrA* gene or the *dksA* gene in the Δ6*ppi* background. Supplementation of casamino acids was necessary, since Δ*dksA* bacteria being auxotrophic do not grow on minimal medium without amino acids. A Δ*hchA* mutation could be introduced into SR18292 on M9 minimal medium. Even on M9 minimal medium Δ(6*ppi hchA*) bacteria form small colonies and further exhibit synthetic growth defects (attenuated growth on LA medium with small colonies after the prolonged incubation of more than 24 h at 37 °C) (Table 3). Among other deletion derivatives of multicopy-suppressing genes, Δ(6*ppi cmk*) transductants were obtained only after the incubation of more than 48 h (Table 3) and hence Cmk is also a limiting factor. The lack of either Hda or Cmk in Δ6*ppi* bacteria could exacerbate DNA replication/synthesis defects and can account for the lethality and synthetic growth defects.

To reinforce the results of the essentiality of the *dksA* gene in Δ6*ppi* bacteria, a strain SR20355 (*dksA*::cm *htrE*::tet) was constructed. This strain served as a donor in bacteriophage P1-mediated transductions to score for co-transduction of *dksA* and *htrE* null alleles in the wild type and its Δ6*ppi* derivative SR18292. The *htrE* gene is >90% linked to the *dksA* gene [18]. The deletion of the *htrE* gene does not confer any growth phenotype at 37 °C in either the wild type or its Δ6*ppi* derivative and could be transduced at the same frequency in both strains (Table 4). However, none of the *htrE*::tet^R^ transductants were found to carry *dksA*::cm in Δ6*ppi*, while >90% of tet^R^ transductants in the wild type were of the (*htrE*::tet^R^ *dksA*::cm) genotype (Table 4). These genetic experiments unambiguously prove that the *dksA* gene is indispensable for the viability of Δ6*ppi* bacteria.

Interestingly, Δ*diaA* and Δ*yjfN* could be transduced into Δ6*ppi* strains without any additional deleterious phenotype. Concerning other candidates, the majority of remaining genes, whose overexpression restores the growth of Δ6*ppi* bacteria, were also found to be required for the optimal growth, since their null combination derivatives conferred a small colony size morphology. However, in such combinations, the transduction frequency in a Δ6*ppi* strain is comparable to that when the parental wild-type strain was used as a recipient. Such a synthetic growth defect was specifically observed, when null alleles of *ariR*,r *pepA*, *ycjW* or *cspC* were introduced into the Δ6*ppi* strain SR18292. However, it should be noted that in MC4100 Δ6*ppi* derivatives (GK4649 or SR18255) the requirement of some of the genes that act as multicopy suppressors is not very stringent (Table 3). For example, a deletion of the *srrA* gene that was conditional as Δ(6*ppi srrA*) confers a cold-sensitive phenotype at 30 °C or below, but is viable at 37 °C, although the colony size is severely diminished on minimal medium. Thus, in summary, we can conclude that functions of DksA, SrrA, MetL and Hda are essential for the survival of Δ6*ppi* bacteria, and Δ*hchA* and Δ*cmk* deletions are very poorly tolerated in Δ6*ppi* derivatives; hence, HchA and Cmk proteins are limiting in Δ6*ppi* bacteria.

### 2.5. Overexpression of Either the dksA Gene or the cmk Gene or the metL Gene Also Restore the Growth of a Δ(dnaK dnaJ tig) Strain

**Figure 5:**
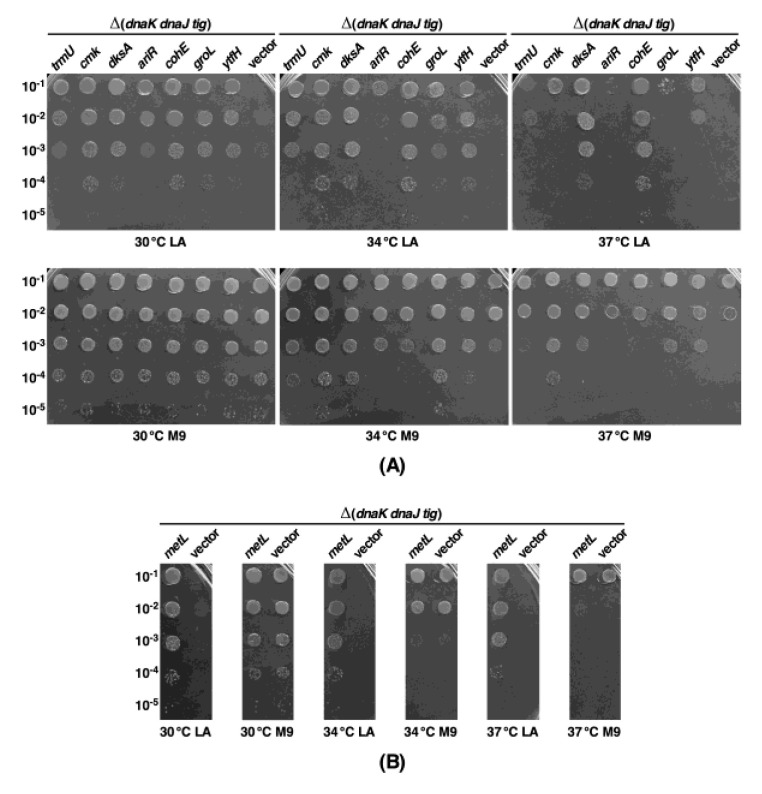
Overexpression of either the *cmk* gene or the *dksA* gene (**A**) or the *metL* gene (**B**) that act as multicopy suppressors of Δ6*ppi* bacteria can restore the growth of a Δ(*dnaK/J tig*) strain under non-permissive growth conditions. Exponentially grown cultures of the wild type and its isogenic Δ(*dnaK dnaJ tig*) derivative transformed with either the vector pCA24N alone or the plasmid expressing the indicated gene were grown in minimal medium at 30 °C. Cultures were washed and adjusted to an optical density OD_600_ of 0.1 and serially spot diluted on M9 minimal medium and LA medium at different temperatures in the presence of 75 μM IPTG.

It has been previously reported that strains that simultaneously lack the ribosome-associated PPI Tig and the chaperones encoded by *dnaK* and *dnaJ* genes exhibit the synthetic lethality on rich medium (LA) at 30 °C and above [19,20]. This phenotype is reminiscent of the synthetic lethality exhibited by Δ6*ppi* bacteria at 30 °C on LA medium [1]. Furthermore, the majority of proteins that aggregate in Δ6*ppi* bacteria are common to strains lacking DnaK/J chaperones [1]. This prompted us to construct Δ(*dnaK dnaJ tig*) derivatives under permissive growth conditions of M9 minimal medium at either 23 °C or 30 °C and seek multicopy suppressors that restore the growth of such a strain on LA medium at 30, 34 or 37 °C. Using the complete library of plasmids from the ASKA collection of single ORFs [13], a Δ(*dnaK dnaJ tig*) strain was transformed and used to identify genes which, when overexpressed upon the addition of 75 μM IPTG as the inducer, can restore the growth of such an attenuated strain under non-permissive growth conditions (LA medium 30, 34 or 37 °C). As the plasmid library contains all the protein-coding genes, this selection for suppressors is saturated. After the retransformation in a Δ(*dnaK dnaJ tig*) strain, plasmids that bred true were sequenced to identify the gene whose overexpression can restore the growth of a Δ(*dnaK dnaJ tig*) strain. This DNA sequence analysis identified fifteen genes (*dksA*, *cmk*, *metL*, *ariR*, *trmU*, *groL*, *ghoS*, *rplJ*, *aegA*, *ytfH*, *glpB*, *glyQ*, *greB, cohE* and *murI*), whose overexpression restored the growth on LA rich medium albeit to different extents at 30 °C. Out of these genes, the most promising multicopy suppressors of a Δ(*dnaK dnaJ tig*) strain on LA medium at 34 °C are: *metL*, *trmU*, *cmk*, *dksA*, *cohE*, *groL* and *ytfH* (Figure 5). However, overexpression of the *ariR* gene can also effectively restore the growth of a Δ(*dnaK dnaJ tig*) strain at 30 °C, but not at 34 °C (Figure 5A). At 37 °C, overexpression of *metL*, *cmk*, *dksA* or *cohE* gene were the only ones that could restore the colony-forming ability of Δ(*dnaK dnaJ tig*) derivative (Figure 5). Among 15 multicopy suppressors, overexpression of the *greB* gene offered only a weaker suppression on LA medium at 30 °C, but not at higher temperatures. Of interest is the re-cloning of *cmk*, *metL*, *dksA*, *groL* and *ariR* as multicopy suppressors of a Δ(*dnaK dnaJ tig*) strain, which in above studies were isolated as multicopy suppressors of the growth defect of Δ6*ppi* bacteria (Figure 3). These results allow us to conclude that there exists an overlap in the function of major chaperones and cytoplasmic PPIs in *E. coli* and both pathways are dedicated to ensure correct folding of proteins in the cell, consistent with the accumulation of several common proteins that aggregate in Δ6*ppi* and Δ(*dnaK dnaJ tig*) derivatives [1]. However, not all multicopy suppressors are common to Δ6*ppi* and Δ(*dnaK dnaJ tig*) bacteria, suggesting some limiting factors are unique to either of such derivatives.

### 2.6. DksA, Cmk and MetL Exhibit the PPIase Activity, Explaining Their Mode of Suppression

**Figure 6:**
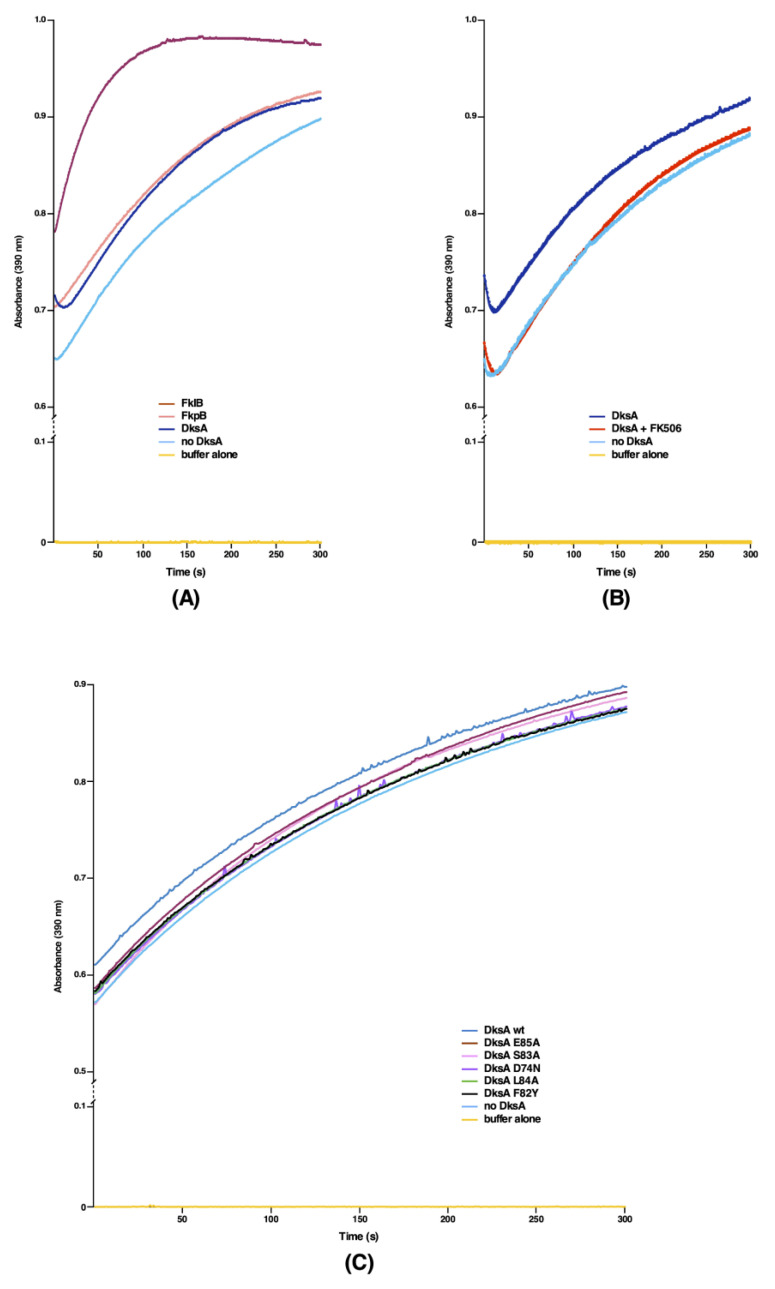
The RNA polymerase-binding protein DksA exhibits a PPIase activity that is comparable to that of FkpB and is inhibited by FK506. The PPIase activity was measured in the chymotrypsin-coupled assay using *N*-Suc-Ala-Ala-*cis*-Pro-Phe-*p*-nitroanilide as the substrate. (**A**) The comparative PPIase activity of FkpB, FklB and DksA proteins. (**B**) DksA was incubated with a two-fold molar excess of FK506 for 5 min at 10 °C prior to the PPIase activity measurement. The PPIase activity of DksA in the presence or absence of FK506, along with uncatalyzed reaction are plotted. (**C**) Measurement of PPIase activity of wild-type DksA and its variants. Proteins were used at a concentration of 5 μM each.

**Figure 7:**
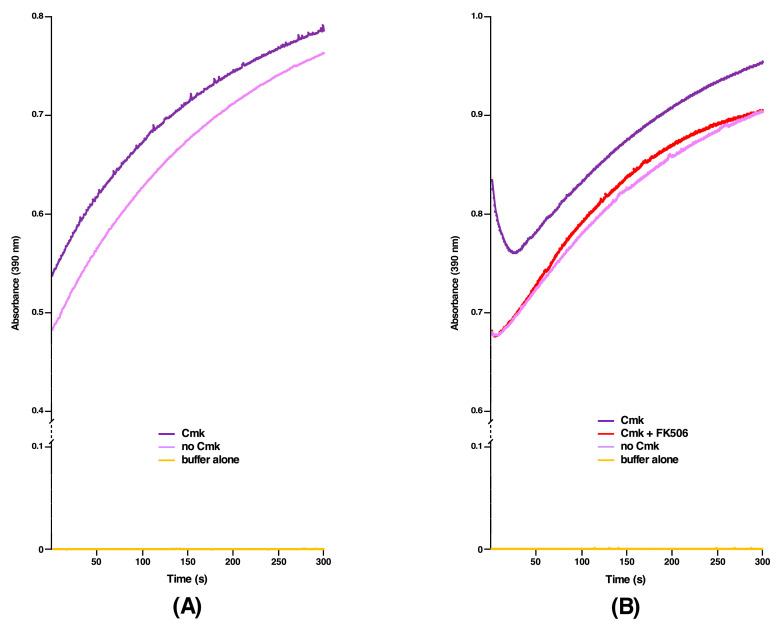
Multicopy suppressor of Δ6*ppi* the cytidylate kinase Cmk exhibits the PPIase activity. (**A**) Measurement of PPIase activity of Cmk in the chymotrypsin-coupled assay, using 5 μM of protein in the assay buffer. (**B**) Measurement of inhibition of the PPIase activity of Cmk by the two-fold molar excess of FK506. The PPIase activity in the presence or absence of FK506 and the uncatalyzed reaction without any enzyme are plotted in the light violet color.

**Figure 8:**
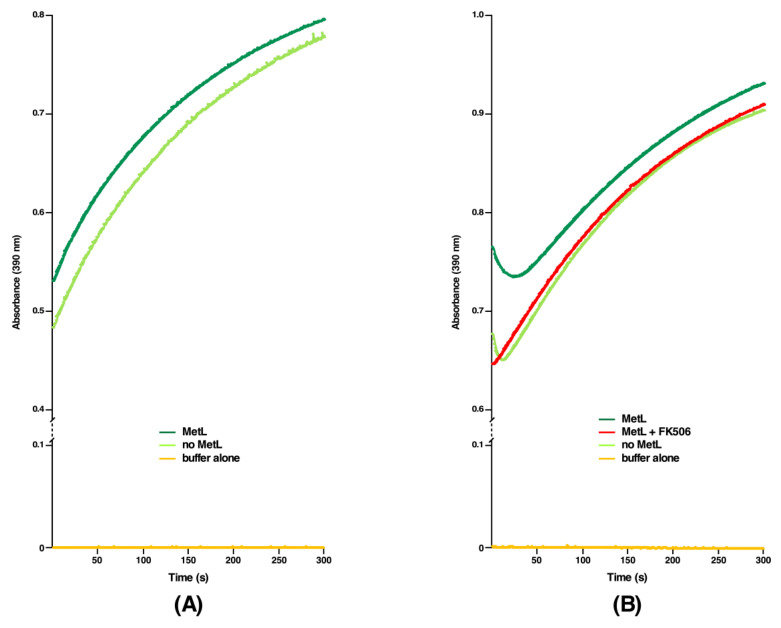
Multicopy suppressor of Δ6*ppi* the asparatokinase kinase II MetL exhibits the PPIase activity. (**A**) Measurement of the PPIase activity of MetL in the chymotrypsin-coupled assay, using 5 μM of protein in the assay buffer. (**B**) Measurement of inhibition of the PPIase activity of MetL by the two-fold molar excess of FK506. The PPIase activity in the presence or absence of FK506 and spontaneous reaction without any enzyme are plotted.

We previously reported that Δ10*ppi* bacteria still retain a weak residual activity, implying that some unidentified PPI(s) could still exist explaining their viability [1]. We rationalized that multicopy suppressors that restore the bacterial growth under non-permissive growth conditions of either a Δ6*ppi* strain or Δ(*dnaK*/*J tig*) bacteria might identify such unknown PPI(s), since the multicopy suppression mechanism can often act by bypassing a requirement of missing factor, if a similar activity is encoded by a suppressing factor. Thus, we purified several proteins, which were identified in above experiments, whose high dosage compensated for the absence of six cytoplasmic PPIs and also restored the growth of Δ(*dnaK*/*J tig*) bacteria. Such purified proteins were tested at the biochemical level for the presence of any PPIase activity in the classical chymotrypsin-coupled assay. All proteins were purified from cell extracts obtained from a Δ6*ppi* strain to prevent contamination from well-characterized highly active six cytoplasmic PPIs. Measurement of PPIase activity revealed that DksA, Cmk and MetL indeed exhibit the PPIase activity (Figure 6, Figure 7 and Figure 8). Among the multicopy suppressors, DksA, Cmk and MetL in high dosage are common to both sets of strains in the suppression of growth defects. However, the PPIase activity of DksA, MetL or Cmk is weaker than that of well characterized PPIs such as FklB with *k*_cat_/*K*_M_ 10^6^ M^−1^ s^−1^ (Figure 6, Figure 7 and Figure 8). Nevertheless, the relative PPIase activity of DksA and FkpB are comparable (Figure 6A). The catalytic efficiency of DksA turns out to be *k*_cat_/*K*_M_ 0.7 × 10^3^ M^−1^ s^−1^ as compared to 1.25 × 10^3^ M^−1^ s^−1^ for FkpB, which is comparable to previously reported activity of FkpB [21]. The relatively weaker PPIase activity of these multicopy suppressing factors can explain why DksA, MetL and Cmk have not been previously identified as PPI enzymes. However, this activity provides a rational explanation for their multicopy suppressing ability. Identification of PPIase activity of DksA can in part explain the previously unknown mechanism of suppression of the growth phenotype of strains lacking DnaK/J chaperones and, in the present study, the restoration of growth of Δ(*dnaK*/*J tig*) and Δ6*ppi* strains and the synthetic lethality exhibited by Δ(*dksA* 6*ppi*), Δ(*cmk* 6*ppi*) and Δ(*metL* 6*ppi*) combinations.

### 2.7. The PPIase Activity of DksA, Cmk and MetL Is Inhibited by FK506

Three distinct families of PPIs are classified on the basis of their specific inhibition. Thus, cyclophilins are inhibited by the cyclosporin A, FKBPs by the FK506 macrolide and parvulins by juglon [22]. However, the sensitivity to the inhibitor is known to vary depending upon the origin of PPIs. Since DksA, Cmk and MetL are not known to belong to any of these three families at structural level, we set out experiments to examine if any of three inhibitors of PPIs can also inhibit our the newly discovered PPIase activity. Interestingly, sequence alignment predicts some similarity in amino acid sequences between DksA and FkpB (see below). Thus, we measured the PPIase activity of DksA, Cmk and MetL proteins in the chymotrypsin-coupled assay in the presence or absence of FK506. Quantification of these data reveals that FK506 can effectively inhibit the PPIase activity of these enzymes. In these experiments, a two-fold molar excess of FK506 was used according to the established protocol [23] in the chymotrypsin-coupled assay with the *N*-Suc-Ala-Ala-*cis*-Pro-Phe-*p*-nitroanilide tetrapeptide as a test substrate to measure the PPIase activity (Figure 6, Figure 7 and Figure 8B). These results thus provide a conclusive evidence showing that DksA, Cmk and MetL exhibit the PPIase activity and hence define a new class of PPIs, whose activity is inhibited by FK506. Thus, the identification of their PPIase activity establishes the molecular basis of multicopy suppression upon their overexpression in Δ6*ppi* bacteria.

### 2.8. DksA, Cmk and MetL Can Catalyze the PPIase-Dependent Refolding of RNase T1

**Figure 9:**
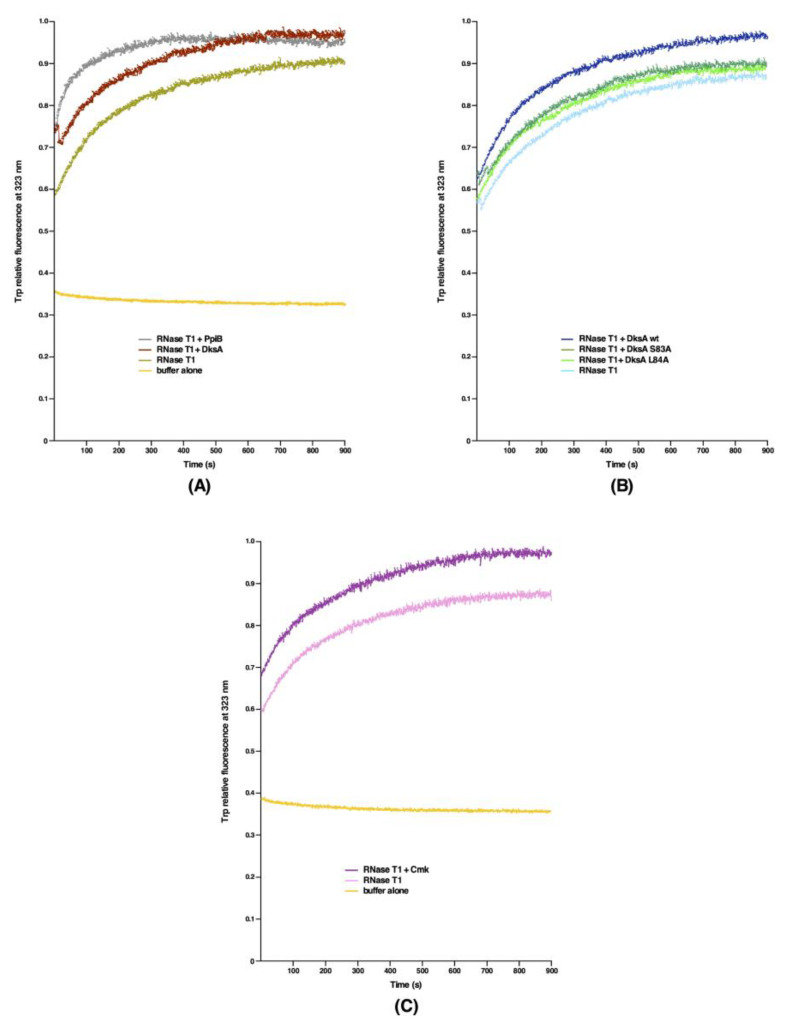
Catalysis of PPIase-dependent refolding of RNase T1. (**A**) The increase in fluorescence at 320 nm is depicted as a function of time of refolding of RNase T1 in the presence of DksA and PpiB. (**B**) Measurement of refolding of RNase T1 in the presence of wild-type DksA and different DksA mutants. (**C**) Refolding of RNase T1 catalyzed by the addition of Cmk as measured by the increase in fluorescence at 320 nm. Reaction when buffer alone is added is depicted in (**A**,**C**).

Next, we measured the PPIase activity in the RNase T1 refolding assay. The RNase T1 enzyme is a well-established protein substrate of PPIs, since the folding of RNase T1 is rate-limited by the *cis/trans* isomerization of two prolyl bonds (Tyr38-Pro39 and Ser54-Pro55) [24]. The PPIase-dependent refolding of RNase T1 after denaturation with 8 M urea was monitored after 40-fold dilution of urea in the presence or absence of DksA, Cmk or PpiB by measuring the increase in tryptophan fluorescence at 320 nm. All of these test proteins contain a single tryptophan residue. The results of such experiments reveal an enhanced emission of fluorescence as compared to buffer alone when RNase T1 was refolded in the absence of any added enzyme (Figure 9). However, the kinetics of refolding in the presence of DksA and Cmk are slower as compared to the PpiB PPI. In these experiments of RNase T1 refolding, we did not include MetL, since it contains multiple tryptophan residues giving a very high signal. Overall, these results are consistent with the presence of the PPIase activity associated with DksA and Cmk in measurements using the chymotrypsin-coupled assay, although to a reduced extent as compared to the PpiB protein, which is not surprising.

### 2.9. Identification of Amino Acid Residues That Are Critical for the PPIase Activity of DksA and Its Multicopy Suppression

**Figure 10:**
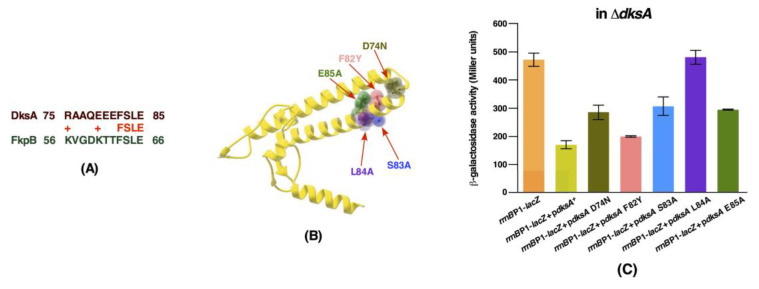
Mutational analysis of amino acid residues critical for the DksA PPIase activity and impact on the transcriptional activity of the *rrnB*P1 promoter. (**A**) Alignment of conserved amino acid residues of DksA and FkpB. (**B**) The position of amino acid residues that were mutated mapping to the coiled-coil tip and adjacent residues in the structure of DksA (PDB 1TJL). (**C**) Measurement of the *β*-galactosidase activity from a single-copy chromosomal *rrnB*P1-*lacZ* fusion in Δ*dksA* derivatives with either the vector alone or its isogenic strains transformed with either the plasmid carrying the wild-type *dksA* gene or plasmids with *dksA* variants. Data are presented from a representative experiment with cultures sampled at OD_600_ 4.0 in the stationary phase. Error bars represent a S.E. of three independent measurements.

The DksA protein is the very well characterized global transcriptional factor, which binds in the secondary channel of RNA polymerase (RNAP). DksA alters transcription by binding to RNAP and allosterically modulates its activity upon amino acid starvation [25]. Strains lacking the *dksA* gene are known to exhibit an auxotrophic growth phenotype and hence are unable to form colonies on minimal medium [26]. Δ*dksA* mutants are also characterized by an increased expression of rRNA promoters such as *rrnB*P1 [26]. Based on the structural analysis and mutagenesis of various residues in the DksA protein, it has been proposed that the coiled-coil domain of DksA inserts into the secondary channel of RNAP and that residues at the tip of the coiled-coil of DksA are important for its activity [27,28]. It has been shown that RNAP β subunit Sequence Insertion 1 (β-SI1) is the binding site for DksA and the tip of DksA interacts with the highly conserved substrate-binding region of the β subunit active site. Thus, we mutated conserved residues in the DksA’s tip motif, particularly those that show limited homology to FkpB of *E*. *coli* (Figure 10A). Specific single amino acid mutations introduced in the coding sequence of the *dksA* gene are D74N, F82Y, S83A, L84A and E85A (Figure 10B). These variants were cloned in the same expression vector, which carries the wild-type Hexa-His-tagged *dksA* gene. The expression in all cases is regulated by the IPTG-inducible P_T5_-*lac* promoter and were examined: (*a*) for the complementation of auxotrophic phenotype of a Δ*dksA* strain; (*b*) if they can restore the growth of either the Δ6*ppi* bacteria or a Δ*dnaK/J* strain when overexpressed; (*c*) the suppression of transcriptional activity of test promoter such as the *rrnB*P1 promoter, whose activity is known to be repressed by overexpression of the wild-type DksA; and (*d*) the relative PPIase activity as compared to the wild-type DksA protein. The results of these experiments are summarized in Table 5. Briefly, overexpression of none of the DksA variants complemented the auxotrophic phenotype of a Δ*dksA* strain, with only F82Y conferring a partial growth on minimal medium (Table 5). Notably, when either the DksA-D74N or the DksA-L84A variant was overexpressed in the Δ*dksA* derivative, they conferred a very tight auxotrophic phenotype on minimal medium. Concerning the ability to repress the *rrnB*P1 promoter activity, overexpression of DksA-L84A variant in Δ*dksA* exhibited the same *β*-galactosidase activity from a single-copy chromosomal *rrnB*P1-*lacZ* fusion as was observed when the vector alone was present in the Δ*dksA* strain (Figure 10C). Furthermore, overexpression of DksA-D74N, DksA-S83A or DksA-E85A caused a significantly milder reduction in the *rrnB*P1-*lacZ* fusion activity as compared to that when the expression of the wild-type *dksA* gene was induced (Figure 10C). Overexpression of only DksA-F82Y variant was found to reduce the *rrnB*P1 promoter activity close to that observed with overexpression of the wild-type *dksA* gene. The activity of *rrnB*P1 promoter has been used as the reporter in several studies and its activity is known to be inhibited by overexpression of the wild-type DksA [27]. Consistent with previous study [27], DksA-D74N variant in our experiments revealed that its overexpression does not complement the auxotrophic phenotype of a Δ*dksA* strain and the activity of the *rnnB*P1 promoter is not repressed to the same extent as when the wild-type DksA is overexpressed. Next, we measured the PPIase activity of various DksA mutants, using purified proteins in the chymotrypsin-coupled assay. Most of the DksA mutants exhibited the relatively reduced PPIase activity as compared to the wild-type DksA (Figure 6C). Interestingly, DksA-F82Y and DksA-L84A substitutions abrogated the PPIase activity of DksA. However, the PPIase activity of DksA-E85A is reduced to a lower extent as compared to other DksA mutants. We further investigated the PPIase activity of DksA-S83A and DksA-L84A variants in the RNase T1 refolding assay. Both mutants exhibited the reduced PPIase activity in this assay as compared to the wild-type DksA (Figure 9C). These results are consistent with the results of PPIase activity measurement of DksA mutants in the chymotrypsin-coupled experiments.

Regarding the suppression of Ts phenotype, none of the variants could restore the growth of a Δ6*ppi* derivative, although the plasmid expressing DksA-E85A mutant protein conferred a leaky phenotype with the appearance of small colonies after a prolonged incubation. Similarly, we also tested the ability of these mutants to suppress the Ts phenotype of a Δ*dnaK/*J strain upon their overexpression. D74N and F82Y substitutions in the DksA protein also totally abrogated the multicopy suppressing ability of Δ*dnaK/*J strain (Table 5). DksA D74N variant has also been reported to be defective in the suppression of Ts phenotype of a Δ*dnaK/*J strain, which supports our results [29]. However, in the previous study, the DksA mutants other than D74N that we describe in this work were not available [29]. Overexpression of S83, L84 and E85 DksA variants also abolished the restoration of growth of a Δ*dnaK/*J strain at 42 °C. Thus, we can conclude that, while the F82Y alteration has the wild type-like ability to repress the *rrnB*P1 promoter activity, its PPIase activity is highly attenuated and does not suppress the Ts phenotype of either a Δ6*ppi* strain or that of Δ*dnaK/*J strain.

Next, the ability of these plasmid-born DksA variants to support the growth of a Δ(*dnaK/J tig*) strain was examined. None of these variants restored the growth of a Δ(*dnaK/J tig*) strain on LA medium at 34 °C upon overexpression, although a leaky growth phenotype at 30 °C was observed for with E85A derivative (Table 5). Taken together, our data show that the PPIase-dependent activity of DksA is required for the suppression of growth defects of either Δ6*ppi* or Δ(*dnaK/J tig*) strains.

### 2.10. The DksA-Mediated Multicopy Suppression of Either Δ6ppi or ΔdnaK/J Mutant Bacteria Requires the Wild-Type Expression of GroL/S and RpoE Essential Proteins

Our work thus far established that overexpression of the secondary channel RNA polymerase-binding protein DksA can efficiently suppress a Ts phenotype of Δ6*ppi* strains and also restore the growth of a Δ(*dnaK/J tig*) strain on LA medium up to 37 °C. The *dksA* gene was originally identified as the multicopy suppressor of a Ts phenotype of the Δ*dnaK*/*J* strain at 42 °C [30]. In the above experiments, we showed that DksA has a PPIase activity, which is necessary for DksA’s multicopy suppression. In the next series of experiments, we identified *trans*-acting factors that are required for DksA’s multicopy suppression. This was achieved by the isolation of Tn*10* transposon insertion mutants in a Δ6*ppi* derivative carrying the *dksA* gene on a plasmid that were unable to grow at 43 °C from a saturated screen of mutants. Those Tn*10* mutants that bred true were transduced into the wild type to eliminate mutations that conferred a Ts phenotype at 43 °C even in the wild-type background. Out of these, 18 Tn*10* insertions were found to be Ts in the wild type, while the remaining were specific to the Δ6*ppi* strain. Among Ts mutants, nine independently isolated mutants carried the Tn*10* insertion in the *degP* gene (Table 6). The *degP* gene is required for the bacterial growth at temperatures above 42 °C and is a member of the *rpoE* regulon [31,32,33]. Interestingly, Δ(*degP dksA*) confers a Ts phenotype above 39 °C, unlike a single Δ*degP* or Δ*dksA*, which are viable under such conditions (Table 6). Concerning the remaining Ts mutants, one of them has the insertion in the *cydA* gene and other has it in the *ftsX* gene (Table 6). The *cydA* gene encodes the cytochrome *bd*-1 ubiquinol oxidase subunit I, which is essential for the bacterial growth at temperatures above 37 °C [34]. Furthermore, a (*cydA*::Tn*10* Δ*dksA*) strain exhibits growth defects even at 30 °C and Δ*dksA ftsX*::Tn*10* was found to be synthetically lethal (Table 6).

**Figure 11:**
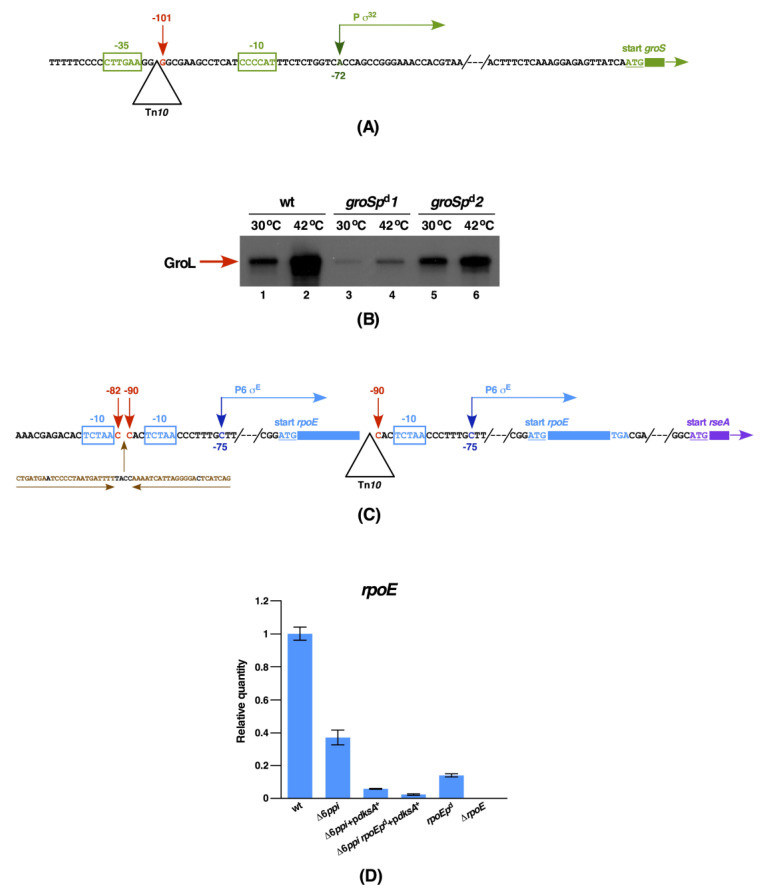
Wild-type expression of GroL and RpoE are required for the DksA-mediated suppression of either Δ6*ppi* or Δ*dnaK/J* bacteria. (**A**) Schematic drawing of the Tn*10* insertion position in the promoter region of the *groS/L* operon. Boxes indicate the location of −10 and −35 heat shock elements recognized by RpoH. (**B**) Western blot analysis of total cell extracts obtained from the wild type (wt) and its two isogenic derivatives carrying *groSp*^d^*1* and *groSp*^d^*2* mutation with or without a heat shock of 15 min at 42 °C. Equivalent amounts of proteins were applied and resolved on 12.5% SDS-PAGE and transferred by Western Blotting. Blots were probed using GroL-specific antibodies and revealed by chemiluminescence. (**C**) The position of the Tn*10* insertion at the nt position −90 in the *rpoE*P6 promoter is indicated. The duplication and the orientation of the intact *rpoE* gene and the disruption of the *rpoE*P6 promoter is shown by an arrow. (**D**) Quantification of the *rpoE* mRNA by qRT-PCR using the total RNA isolated from the isogenic wild type (wt), SR18292 (Δ6*ppi*), SR20561 (Δ6*ppi* + p*dksA*^+^), GK5165 (Δ(6*ppi rpoEp*^d^) + p*dksA*^+^), GK5347 (wt *rpoEp*^d^) and SR8691 (Δ*rpoE*) strains. For RNA isolation, cultures were grown under permissive growth conditions of M9 at 37 °C.

The remaining mutants that do not confer a Ts phenotype in the wild-type background identified 15 genes whose products/amounts of encoded proteins are crucial for the DksA-mediated suppression. Out of these, two insertions mutations identified GK5109 and GK5578 strains have Tn*10* inserted at the identical position in the promoter region of the essential chaperonin-encoding *groSL* operon. This Tn*10* insertion in the *groSL* operon is located 3 nt downstream of the −35 heat shock promoter element, which is 101 nt upstream of the translational initiation codon (Figure 11A). Although both insertions abolish the DksA-mediated suppression of Δ6*ppi*, the insertion in GK5109 strain also confers the small colony morphology at 40 °C and the inability to grow at 43 °C in the wild type as compared to the nearly normal growth of GK5578 derivatives. To address differences in growth properties exhibited by two strains with the identical position of Tn*10* insertion in the *groSL* promoter, the amount of GroL was measured from total cell extracts. Thus, cultures of wild-type bacteria and its derivatives GK5109 and GK5578 (Δ6*ppi* + p*dksA*) *groS101::*Tn*10* were grown under permissive conditions and subjected to a 15-min heat shock treatment at 42 °C. The relative abundance of GroL was analyzed using Western blot technique. Such experiments revealed that the amount of GroL is significantly reduced in the strains with the Tn*10* insertion in the promoter region of *groSL* and the induction of GroL at higher temperature is impaired, which is consistent with the disruption of −35 element of RpoH-regulated heat shock promoter (Figure 11B). Furthermore, the amount of GroL is higher in the strain GK5578 as compared to the strain GK5109, explaining the differences in the growth phenotype of these strains (Figure 11B). Thus, transcription of the *groSL* operon in these strains is independent of the RpoH-regulated heat shock promoter and is driven from the promoter generated by the insertion element. This interesting difference with the similar position of insertion elements in the promoter region synthesizing different levels of GroL and the degree of suppression can be explained by the potential creation of promoter of different strength within the insertion element that drive *groSL* transcription. Such differences arising due to changes in transcription due to identical insertion elements in the promoter region is not without precedence and has been reported previously within the *groSL* promoter [35]. Significantly, these *groSL* promoter mutations also abolished the multicopy suppression of a Δ*dnaK/J* mutation (Table 6). The isolation of mutations in the promoter region of the *groSL* operon that allow their constitutive expression with the dysfunctional heat shock promoter suggests that at high temperature DksA overexpression cannot suppress either a Δ6*ppi* or a Δ*dnaK/J* mutation, since GroL and GroS proteins are not heat shock induced and hence they become limiting. Thus, the DksA multicopy suppression requires wild-type levels of GroL/S chaperonins and their heat shock induction.

**Figure 12:**
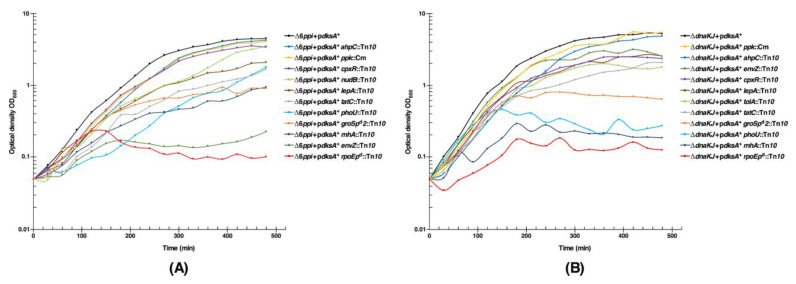
Wild-type levels of RpoE, GroL/S and intact copies of RNase H, TatC and LepA are required for the DksA-mediated suppression of Ts growth of either Δ6*ppi* or Δ*dnaK/J* strains. (**A**) Isogenic cultures of Δ6*ppi* + p*dksA*^+^ and its derivatives with either the Tn*10* insertion or deletion were grown overnight in M9 medium at 37 °C. Culture were adjusted to OD6_00_ of 0.05 in 10 mL of prewarmed LB medium at 43.5 °C. Aliquots of samples were drawn at different intervals and the bacterial growth monitored by measuring OD_600_. (**B**) Isogenic cultures of Δ*dnaK/J* + p*dksA*^+^ and its derivatives with gene disruptions were grown in M9 medium at 30 °C and analyzed for the bacterial growth in LB medium at 41 °C by measuring OD_600_. Data presented are from a representative experiment.

Two other Tn*10* insertions of significance were located at the position 90 nt upstream of the translational initiation codon of the essential *rpoE* gene that totally abolished the multicopy suppression by the *dksA* gene (Table 6, Figure 11B). This Tn*10* insertion is located 3 nt upstream of the −10 *rpoE*P6 promoter element. This *rpoE* promoter is the most proximal promoter, which is transcribed by Eσ^E^ [15]. As the *rpoE* gene is essential for the viability of *E. coli*, we wondered if its expression is abolished by this Tn*10* insertion. DNA sequencing of the *rpoE* gene and its adjoining region revealed that an extra copy of the intact *rpoE* gene from −90 nt until its end had been duplicated at the Tn*10* insertion site in the same orientation (Figure 11C). Further analysis of the *rpoE* gene expression by quantitative reverse transcription PCR (qRT-PCR) revealed that this Tn*10* insertion leads to a 10-fold reduction of its transcription, which is further reduced when the *dksA* gene is overexpressed (Figure 11D). As a control, total mRNA from the Δ*rpoE* strain SR8691 was included in this qRT-PCR experiment, which did not show any *rpoE*-specific amplification products (Figure 11D). Thus, the DksA-mediated multicopy suppression requires full RpoE functionality. This allele for brevity is called *rpoEp*^d^ (down mutation). Consistent with our previous results, a Δ6*ppi* strain exhibits a reduction in the expression of the *rpoE* gene under permissive growth conditions [1] (Figure 11D) and could hence become severely limiting in the presence of *rpoEp*^d^ mutation. Interestingly, the *rpoEp*^d^ mutation also abrogated the multicopy suppression by the *dksA* gene of the Ts phenotype of a Δ*dnaK/J* strain at either 40 or 42 °C (Table 6 and Figure 12). Taken together, the DksA-mediated multicopy suppression of either a Δ6*ppi* or a Δ*dnaK/J* strain requires the wild-type level expression of the *rpoE* gene and *groS*/*L* genes.

### 2.11. The DksA-Mediated Multicopy Suppression Requires Cell Envelope Homeostasis, the Genome Integrity, the Ribosome Assembly, Translocation of Folded Proteins and Factors Combating Oxidative Stress

Mapping and characterization of remaining Tn*10* insertions that prevent the multicopy suppression by DksA can be grouped in four functional categories: (*i*) the cell envelope composition and its integrity (*cpxR*, *envZ*, *tolA*, *mrcB* and *clsA*); (*ii*) the genome integrity and repair (*rnhA* and *nudB*); (*iii*) translation and transport systems (*lepA* and *tatC*); (*iv*) phosphate uptake and phosphate sensing system (*phoU* and *pstS*); and (*v*) genes whose products are related to the oxidative stress responsive pathway (*ahpC* and *oxyR*) (Table 6). This mutagenesis is saturated, since Tn*10* insertions in some of these genes were obtained multiple times (three each in *envZ* and *tolA* and two each in *clsA* and *ahpC*). Furthermore, the introduction of *rnhA*::Tn*10*, *lepA*::Tn*10*, *phoU*::Tn*10*, *ahpC*::Tn*10*, *oxyR*::Tn*10, tolA*::Tn*10*, *tatC*::Tn*10* and *envZ*::Tn*10* also abrogated the multicopy suppression of Δ*dnaK/J* by DksA at either 40 °C or 42 °C on LA medium, although to a variable extent (Table 6). Consistent with these results, a synthetic growth phenotype was observed with Δ(*dksA degP*), Δ(*dksA lepA*), Δ(*dksA tatC*), Δ(*dksA rnhA*) and Δ(*dksA phoU*) combinations (Table 6).

To validate the requirement of above-mentioned genes, whose products are required for the DksA-mediated multicopy suppression of either a Δ6*ppi* or a Δ*dnaK/J* derivative, kinetics of the bacterial growth was measured in aerobically shaken cultures. Isogenic bacterial cultures of SR20561 (Δ6*ppi* + p*dksA*^+^) alone and its derivatives with the Tn*10* insertion in the specific gene were grown under the permissive growth condition of M9 minimal medium at 37 °C and shifted to LB medium at 43.5 °C. Measurement of optical density OD_600_ at different intervals confirmed that there exists an acute requirement for the adequate expression of either the *groSL* operon or the *rpoE* gene for the DksA-mediated multicopy suppression of Δ6*ppi* bacteria (Figure 12A). A similar dependence for the presence of intact RpoE and GroL/S was found for the multicopy suppression by DksA of a Δ(*dnaK dnaJ*) derivative in the liquid culture (Figure 12B). However, the requirement for the intact *rpoE* gene is more stringent as the *rpoEp*^d^ mutation in the SR20561 (Δ6*ppi* + p*dksA*^+^) background was much more severely affected. It should be noted that for the growth analysis with the Tn*10* insertion in the *groS* promoter we used the *groSp^d^2* derived strain, since this mutation does not confer the Ts phenotype per se as the *groSp^d^1* mutation (Figure 11B and Figure 12 and Table 6).

Concerning the quantitative growth measurement of other Tn*10* insertions in the SR20561 (Δ6*ppi* + p*dksA*^+^) background, the order of severity of the growth inhibition after the *rpoEp*^d^ mutation is *envZ*::Tn*10* > *rnhA*::Tn*10*, *groSp^d^2*::Tn*10*, *phoU*::Tn*10*, *tatC*::Tn*10* and *lepA*::Tn*10* at 43.5 °C in LB medium (Figure 12A and Supplementary Table S2). Surprisingly, the Tn*10* insertion in the c*pxR* gene, the *ahpC* gene or the *nudB* gene exhibited only a minor detrimental growth effect in the liquid culture (Figure 12) and hence the multicopy suppression by the *dksA* gene does not absolutely require such genes in all growth conditions. Similarly, no significant growth inhibition in the liquid culture was observed, when *clsA*::Tn*10* or *mrcB*::Tn*10* were introduced in the strain SR20561 (Δ6*ppi*+p*dksA*^+^). As *tolA*::Tn*10* derivatives in (Δ6*ppi* + p*dksA*^+^) grew quite poorly in liquid culture, such a strain was not analyzed for growth properties. In summary, we can conclude that, besides a requirement for RpoE and GroS/L chaperonins, the genome integrity (RnhA), the outer membrane protein homeostasis (EnvZ), the balanced translational process (LepA), the Tat-dependent translocation of folded proteins and appropriate regulation of phosphate regulon are required for the DksA-mediated multicopy suppression of Δ6*ppi* bacteria.

For the measurement of growth of Δ*dnaK/J* + p*dksA*^+^ derivatives with Tn*10* insertions in various genes that block the DksA-mediated suppression of Δ6*ppi*, all such Tn*10* insertions were transduced in a Δ*dnaK*/*J* strain SR20733. Isogenic cultures were grown under permissive growth condition (M9 minimal medium at 30 °C) and shifted to LB medium at either 40.5 or 41 °C. Data are presented from a representative set of experiments from growth conditions at 41 °C. Measurement of optical density OD_600_ at different growth intervals and determination of growth rate confirmed the inability of DksA-mediated suppression with the *rpoEp*^d^ mutation, with the order Ts growth inhibition following the *rpoE* mutation as *rnhA*::Tn*10* > *phoU*::Tn10, *groSp^d^2*::Tn*10*, *tolA*::Tn10, *tatC*::Tn*10* and *lepA*::Tn*10* (Supplementary Table S2). In contrast to a requirement for the EnvZ function in Δ6*ppi* bacteria, no such dependence was observed in the Δ*dnaK/J* background for the suppression by DksA. Again, no stringent requirement was observed for the presence of either the *cpxR* gene or the *ahpC* gene in the growth in the liquid culture (Figure 12B). Thus, besides a requirement for RpoE and GroL/S, the genome integrity maintenance by RnhA, protein synthesis/translocation (LepA and TatC), the outer membrane integrity (TolA) and phosphate sensing/regulation of phosphate uptake are needed for the multicopy suppression by DksA of either Δ6*ppi* or Δ*dnaK/J* derivatives.

### 2.12. The ppk Gene Encoding Polyphosphate Kinase Is Not Required for the DksA-Mediated Multicopy Suppression of a Δ6ppi Strain and Only a Marginal Requirement for a Δ(dnaK/J) Derivative

Next, we addressed, if the *ppk* gene encoding the polyphosphate kinase is required for the DksA-mediated multicopy suppression. This experiment was included, since Ppk has a chaperone-like function and was identified as a substrate of PpiC and FklB PPIs [1]. Thus, a non-polar deletion of the *ppk* gene was constructed and introduced into SR20561 (Δ6*ppi* + p*dksA*^+^) and SR20733 (Δ*dnaK/J* + p*dksA*^+^) strains under permissive growth conditions on M9 minimal medium and examined for growth properties. On LA medium, Δ(6*ppi ppk*) + p*dksA*^+^ grew nearly to the same extent as its parental strain SR20561 (Δ6*ppi* + p*dksA*^+^) at 43.5 °C (Table 6). Similarly, in the liquid culture, the Δ(6*ppi ppk*) + p*dksA*^+^ derivative grew nearly to the same level as its isogenic strain SR20561 (Δ6*ppi* + p*dksA*^+^) at 43.5 °C, with only a minor growth reduction (Figure 12A). These results thus establish that the *ppk* gene is not essential for the DksA-mediated multicopy suppression of a Δ6*ppi* derivative. Regarding the comparative growth of a Δ(*dnaK/J ppk*) + p*dksA*^+^ derivative with its isogenic parental strain SR20733 (Δ*dnaK/J* + p*dksA*^+^) on LA medium, a similar growth was obtained at 40 °C on LA medium (Table 6). However, on LA medium at elevated temperature of 42 °C, which is the uppermost limit for the growth of a Δ*dnaK/J* + p*dksA*^+^ strain, no viable single colonies were obtained when a Δ(*dnaK*/*J ppk*) + p*dksA*^+^ derivative was tested (Table 6). Furthermore, only a minor growth difference was obtained between SR20733 (Δ*dnaK/J* + p*dksA*^+^) and SR22300 Δ(*dnaK*/*J ppk*) + p*dksA*^+^ derivative as compared to the drastic growth inhibition when Tn*10* insertions in *lepA*, *rnhA*, *tatC*, *envZ* genes or *rpoEp*^d^ mutation were examined (Figure 12B). Thus, we can conclude that Ppk is not required for the DksA-mediated multicopy suppression of Δ6*ppi* bacteria and only a minor growth reduction is observed when the *ppk* gene is absent when the *dksA* gene is overexpressed in strains lacking *dnaK*/*J* at temperatures around 42 °C, but not at either 40 or 40.5 °C on LA medium or up to 41 °C in liquid culture.

## 3. Discussion

PPIs are universally conserved enzymes that catalyze the *cis*/*trans* isomerization of peptide bonds that precede the proline amino acid. PPIs accelerate this rate-limiting step in protein folding of the *cis*/*trans* isomerization of the peptide bond by a factor of 10^3–^10^6^ [36,37]. PPIs have been classified into three families depending on the sensitivity to specific inhibitors. The cytoplasm of *E*. *coli* contains six such enzymes representing all three families. However, their substrates were only recently described [1]. Although individually all six cytoplasmic PPIs are non-essential for bacterial viability, collectively the removal of all such enzymes confers synthetic lethality, which is manifested by the inability of such Δ6*ppi* bacteria to grow on rich medium and the temperature sensitivity (Ts) on rich as well as minimal medium [1]. In this work, we further characterized various growth defects of Δ6*ppi* bacteria and show that they exhibit highly pleiotropic phenotypes. These include the inability to grow under fast-growing conditions of rich medium, and the sensitivity to: (*i*) DNA-damaging agents such as nalidixic acid indicating that the genome integrity of Δ6*ppi* bacteria is compromised; (*ii*) cell envelope-destabilizing agents; and (*iii*) the exposure to ethanol and the inability to grow on minimal medium, when glycerol was used as the sole carbon source. Taking advantage of such growth defects, multicopy suppressors that restore the viability of Δ6*ppi* bacteria under non-permissive growth conditions were isolated to identify factors that are limiting for the viability of such bacteria. We also reasoned that potentially some multicopy suppressors could compensate growth defects of Δ6*ppi* bacteria by possessing a PPIase activity, explaining the presence of the residual PPIase activity in strains lacking all *E*. *coli*’s known PPIs.

Isolation of multicopy suppressors identified cellular factors that are limiting in Δ6*ppi* bacteria and the reasons for the essentiality of PPIase activity. Consistent with the role of PPIs in protein folding, several prominent suppressors identified are: (*i*) major cytosolic chaperones; (*ii*) transcriptional factors such as DksA or SrrA; (*iii*) antitoxin MqsA and toxin Hha; (*iv*) the modulator of the proteolytic activity; (*v*) those involved in combating oxidative stress/acid resistance or detoxification of cysteine; (*vi*) the aspartokinase II MetL involved in the amino acid synthesis; and (*vii*) factors that modulate the chromosomal replication initiation via regulating the activity of initiator protein DnaA (DiaA and Hda). Isolation of DnaK/J and GroL/S as multicopy suppressors is consistent with our previous identification of several proteins that are substrates of these chaperones and PPIs [1]. Thus, PPIs and the molecular chaperone network are together required for folding of cellular proteins. Other multicopy suppressors such as transcriptional factors could act by the enhancing transcription of genes, whose products are needed to maintain proteostasis or by the repressing transcription of genes, whose products become toxic in Δ6*ppi* bacteria.

Next, we systematically removed individual genes, which in multicopy suppressed growth defects of Δ6*ppi* bacteria, other than *dnaK*/*dnaJ* and *groL*/*groS* genes. These studies revealed that Δ6*ppi* bacteria require DksA, MetL, SrrA and Hda for their viability as their deletion combinations in the Δ6*ppi* background turns out to be lethal. Since Δ6*ppi* bacteria exhibit the sensitivity towards DNA-damaging agents could be one of the reasons for the synthetic lethality with Δ*hda*. In support of these data, it has been reported that *hda* mutations increase the cellular level of ATP-DnaA and cause the over initiation of replication, which results in the inhibition of cell division and cell growth [38]. This can help to explain the synthetic lethality of Δ(6*ppi hda*) combination. A requirement for survival of Δ6*ppi* bacteria on the presence of Cmk was also observed, since Δ(6*ppi cmk*) exhibited exacerbated synthetic growth defects. The function of SrrA is at present unknown. Homology searches predict it to be a putative transcriptional factor with the predicated N-terminal PAS domain and the C-terminal helix-turn-helix motif. As overexpression of the *srrA* gene not only restores the growth of Δ6*ppi* strains on rich medium at elevated temperatures, but also can suppress the Nal sensitivity, we believe that SrrA plays an important role in stress response.

Given some of the phenotypes exhibited by Δ6*ppi* and Δ(*dnaK/J tig*) bacteria such as the inability to grow on LA medium and the vast number of proteins that aggregate in such mutants show an overlap, we also undertook the multicopy suppressor approach with a Δ(*dnaK/J tig*) strain. Quite significantly, we identified a set of genes such as *dksA*, *cmk*, *metL*, *groL*, *ariR* whose overexpression resulted in the restoration of growth of a Δ(*dnaK/J tig*) strain, although to different degrees depending on the growth temperature. As these genes were already identified as multicopy suppressors of Δ6*ppi* bacteria suggested that their mechanism of suppression could operate by providing a function related to the protein folding process. The MetL aspartokinase II is a bifunctional enzyme known to be required for the synthesis of amino acids such as a threonine, lysine and methionine [39]. However, overexpression of the *metL* gene offers a better suppression on LA medium than on M9 medium for both either Δ6*ppi* or Δ(*dnaK/J tig*) bacteria arguing an additional function other than its requirement in the amino acid biosynthesis pathway. Using a different plasmid DNA library, an independent study also reported that overexpression of the *dksA* gene can rescue growth defects of a Δ(*dnaK/J tig*) strain [40]. However, in that study [38], certain other genes whose products are involved in metabolic processes such as *ldhA*, *lpd*, *talB*, *tpx*, *ackA*, *nagB, pykF* and *sseA* were identified as multicopy suppressors of a Δ(*dnaK/J tig*) strain that restore the growth on rich medium above 33 °C. Thus, we used a controlled overexpression of such genes [40], whose products are involved in metabolic functions, using plasmids from the ASKA library [13]. However, their overexpression did not restore the growth of a Δ(*dnaK/J tig*) strain and hence those results could not be validated. This explains why such genes were not identified in our multicopy selection using the complete genomic library that could identify *dksA*, *metL*, *groL*, *cmk*, *cohE* and *ariR* as validated multicopy suppressors.

Based on the identification of multicopy suppressors that restore the growth of either Δ6*ppi* or Δ(*dnaK/J tig*) bacteria, two most significant findings of this work are: (*a*) the identification of three new PPIase activities that can explain in part the pathway of suppression; and (*b*) the identification of factors that are required for the DksA-mediated multicopy suppression. We rationalized that products of multicopy suppressors could identify function(s) missing in strains that are compromised in protein folding, when PPIs are absent. Thus, we purified products of all major 15 multicopy suppressors other than GroL/S and DnaK/J protein folding chaperons. Among these DksA, Cmk and MetL proteins exhibited a PPIase activity in the chymotrypsin-coupled assay and in the protein substrate RNase T1, whose folding is limited by the *cis/trans* proline isomerization. Furthermore, this PPIase activity of DksA, Cmk and MetL could be inhibited by FK506 establishing them as bona fide PPIs. It should be noted that, although the PPIase activity of DksA is 1000-fold less than classical PPIs such as FklB [*k*_cat_/*K*_M_ 0.7 x 10^3^ M^−1^ s^−1^ for DksA vs. *k*_cat_/*K*_M_ 10^6^ M^−1^ s^−1^ for FklB (Figure 6A)], it is still comparable to the PPIase activity of FkpB—a well characterized PPI with the low PPI activity with several substrates [1,21]. However, more experiments will be required to identify the binding affinity of these proteins with FK506, the contact regions where FK506 can bind and exact residues involved in catalysis.

Although on the basis of sequence homology none of these proteins exhibit the significant structural homology to any of three classes of PPIs, a segment in DksA (amino acid residues 75–85) located near the coiled-coil tip that protrudes in the secondary channel of RNAP aligns with FkpB amino acid residues 56–66. DksA and other secondary channel binding proteins such as GreA and GreB have a similar fold in the coiled-coil tip domain, but they are not similar at the amino acid sequence level [41,42]. Interestingly, GreA and GreB C-terminal domains exhibit the structural similarity with FKBPs [43]. However, this C-terminal domain is absent in the sequence of DksA. Based on the amino acid alignment of DksA with FkpB and the importance of coiled-coil tip and nearby residues, we constructed D74N, F82Y, S83A, L84A and E85A substitutions, purified such proteins and tested their PPIase activity. All such variants other than E85A exhibited the reduced PPIase activity and did not complement the auxotrophic phenotype of Δ*dksA* and were unable to restore the growth of either a Δ6*ppi* or a Δ*dnaK/J* or a Δ(*dnaK/J tig*) derivative. However, D74N and L84A are much tighter as far as the auxotrophic phenotype is concerned. Quite interestingly, the repression of the *rrnB*P1 promoter activity by DksA variants does not match with the multicopy suppression phenotype of Δ6*ppi* bacteria. For example, the F82Y variant represses the *rrnB*P1 promoter activity quite similar to the wild-type DksA (Figure 10C), but it has the lowest PPIase activity and also cannot rescue the growth of either Δ6*ppi* or Δ*dnaK/J* bacteria (Figure 6C and Table 5). Such results suggest that the PPIase activity of DksA matches with its ability to perform the rescue of growth defects of Δ6*ppi*. It is of interest that D74N variant of the DksA protein has been very well-characterized [27,28]. DksA D74N mutant protein has been shown to bind RNA polymerase, but was defective in the transcription initiation [27,28] and unable to suppress the Ts phenotype of a Δ*dnaK/J* mutant [29]. It has been suggested that the D74 amino acid residue in the coiled-coil tip of DksA is important for the substrate binding of the active site of RNA polymerase and electrostatic charge of carboxyl group is critical for D74 function [27,28]. Amino acid residues R678 and R1106 in the β subunit of RNAP are presumed to interact with D74 of DksA [27,28]. DksA L84 and DksA F82 are also predicted to interact with β’ subunit and L84 could be cross-linked to R744-M747 amino acid residues [28] and hence can impact DksA function, explaining the inability of DksA F82Y and L84A to suppress growth defects of a Δ6*ppi* bacteria. At present, however, we do not know if the PPIase activity is directly required for the DksA-RNAP interaction and future studies are needed to address this vital issue. In the structure of RNA polymerase α, β and β’ subunits, *cis* proline residues are known to be present [1] and DksA’s PPIase activity may be required for the isomerization of their prolyl residues. Furthermore, DksA could prevent the aggregation of RNAP major subunits in Δ6*ppi* and Δ*dnaK/J* mutants, which are known to aggregate in such strains. Thus, finding the natural cellular substrates of DksA, Cmk and MetL requires further extensive studies.

DksA has been structurally and functionally well-studied, yet the mechanism of suppression of the Ts phenotype of Δ*dnaK/J* has remained elusive [29]. As DksA turned out to be an effective suppressor of Ts phenotype of Δ6*ppi* and Δ(*dnaK/J tig*) bacteria, and exhibits a PPIase activity, we also identified genes whose products are required for its multicopy suppressor phenotype. Thus, we showed that the DksA-mediated multicopy suppression is abolished when the expression of either RpoE sigma factor or chaperonins (GroL/S) are reduced. Thus, a unique mutation *rpoEp*^d^ that caused a disruption of the promoter element of the *rpoE* gene, even though its structural gene was duplicated, abolished the suppression by DksA not only of Δ6*ppi* but also of Δ*dnaK/J* bacteria. RpoE responds to the cell envelope stress and positively regulates the expression of many genes, whose products are involved in folding and assembly of outer membrane proteins (OMPs) and sRNAs that act in the feedback mechanism to repress synthesis of major components of the cell envelope such as OMPs and lipoproteins to maintain the cellular homeostasis [44]. Examination of transcription of the *rpoE* gene revealed that its expression is repressed in Δ6*ppi* at permissive growth conditions and is further reduced in a *rpoEp*^d^ background. Thus, when the *dksA* gene is expressed from a plasmid, it further decreases the *rpoE* transcription that could lead to abolishing of the DksA-mediated restoration of growth. This also explains the isolation of mutations in *envZ* and *tol*A genes that abolish the suppression of Δ6*ppi* and Δ*dnaK*/*J* bacteria at high temperature by DksA. Since *tolA* mutants exhibit the enhanced RpoE induction and hypervesiculation [45,46] is consistent with the DksA-mediated suppression being sensitive to hyperinduction of the RpoE regulon. Thus, connection between the DksA-mediated restoration of growth of either Δ*dnaK*/*J* or Δ6*ppi* is highly dependent on maintenance of homeostasis of extracytoplasmic components and balanced signaling of envelope stress. Supporting this pathway is also isolation of the *cpxR*::Tn*10* insertion that abolish the multicopy suppression by DksA. The activation of CpxR/A two-component system induces the expression of several periplasmic folding factors including *degP, ppiD* and *dsbA* [47,48]. The importance of GroL/S abundance for survival of either Δ6*ppi* or Δ(*dnaK/J tig*) derivatives is further highlighted in this work, since (*a*) *groS*/*L* overexpression suppresses growth defects of such strains and (*b*) the isolation of *groSL* promoter mutations reducing transcription of this operon also abolished the multicopy suppression by DksA. Interestingly, the isolation of Tn*10* insertion located 101 nt upstream of the initiation codon is similar to the previously isolated insertion element as a suppressor mutation of the *rpoH* deletion that creates a new promoter, allowing the constitutive expression of the *groSL* operon [35]. It is well established that *groS*/*L* genes are essential for bacterial growth under all growth conditions [49,50].

The isolation of *rnhA*::Tn*10* that totally abolished the suppression by DksA of either Δ*dnaKJ* or Δ6*ppi* is in line with the DksA’s important role in DNA damage repair [11]. The RnhA function is crucial in the removal of RNA-DNA hybrids and prevention of the lethality due to the R-loop formation [51]. Thus, RnhA and DksA are both important in this process of maintaining the genomic integrity and this appears to be impaired in Δ6*ppi* bacteria. In support of these conclusions, we have shown that Δ6*ppi* bacteria exhibit the sensitivity towards DNA-damaging agents such as nalidixic acid—a phenotype shared with *rnhA* and *dksA* mutants. This role of DksA for DNA repair system draws further support, since overexpression of the *dksA* gene can suppress the sensitivity of Δ6*ppi* bacteria to Nal. It is likely that the SrrA-mediated suppression of Nal sensitivity operates via the same pathway as DksA. The suppression by DksA was also found to require the functional Tat system of protein translocation. The insertion mutation in the *tatC* gene will abrogate the export of folded proteins that require the Tat system. TatC is the first protein to interact with the N-region of Tat signal peptide [52]. Interestingly, SlyD and DnaK have been shown to bind Tat signal sequences [53,54] and the Tat system could recruit PPIs in assisting translocation. Other prominent insertion defines the LepA requirement for the DksA suppression. Consistent with our results, a synthetic growth defect of Δ*lepA* and Δ*dksA* has also been reported [55]. The *lepA* gene encodes an elongation factor EF4 GTPase with a role in the biogenesis of 30S subunit of ribosomes and the translational initiation [56]. These results explain abrogation of DksA-mediated suppression when either the translational apparatus or the genome integrity are compromised.

We also addressed whether there is any requirement for the Ppk polynucleotide kinase for the DksA-mediated multicopy suppression, since it was identified as one of the prominent co-eluting proteins during the purification of PPIs [1]. However, Δ(6*ppi ppk*) + p*dksA^+^* and Δ6*ppi* + p*dksA^+^* derivatives grew nearly to the same extent at 43.5 °C either on LA or LB medium, ruling out any requirement for the Ppk function. Even Δ(*dnaK/J ppk*) + p*dksA^+^* and Δ*dnaK/J* + p*dksA^+^* grew nearly similarly up to 41.0 °C. However, no viable colonies were observed at 42 °C, which is the temperature limit at which DksA can exert the suppression of Ts phenotype of a *dnaK/J* deletion, suggesting only a minor requirement. During this manuscript preparation, it was reported that Ppk is required for the DksA-mediated restoration of growth a *dnaK* Ts mutant strain at 40.5 °C [57]. However, we did not observe any such requirement at the same growth temperature. We showed that a *phoU*::Tn*10* insertion abolishes the DksA-mediated multicopy suppression, which further reinforces the notion that Ppk may not be needed for the multicopy suppression phenotype, since it has been reported that *phoU* mutants have elevated levels of polyphosphates [58].

It is intriguing why overexpression of the *dksA* gene does not suppress growth defects of Δ6*ppi* bacteria at 37 °C but shows a good suppression at elevated temperatures in contrast to the suppression of Δ(*dnaK*/*J tig*) at 30 or 37 °C. This suggests that the transcription induction or repression of specific gene(s) at elevated temperatures is a requirement for the DksA-mediated suppression for Δ6*ppi* bacteria. At high temperature, mainly RpoH and RpoE regulons are induced. Hence, we propose a model that the DksA suppression depends on the heat shock induction of GroL/S chaperonins and the RpoE induction, consistent with the isolation of transposon insertions in their promoter regions that renders their transcription independent of heat shock promoters and abolish the multicopy suppression by DksA. As DnaK/J negatively regulate the heat shock response, Δ(*dnaK*/*J tig*) mutants have already elevated levels of GroL/S chaperonins. Consistent with this idea is the isolation of GroL as a multicopy suppressor of (Δ(*dnaK*/*J tig*) bacteria. The DksA-mediated suppression could be to maintain proper folding in the cytoplasm and extracytoplasm via the transcriptional regulation, sensing oxidative stress and due to its PPIase activity. The regulation of extracytoplasmic stress response could be the essential pathway, since insertions that could elevate the RpoE activation abolish the DksA suppression and DksA was also shown to suppress a lethal phenotype of null mutation of the gene encoding the periplasmic Prc (Tsp) protease [59]. The identification of common insertion mutations that abolish the suppression by DksA in strains lacking either PPIs or DnaK/J chaperones strongly suggests that various aspects of cellular defects are presumably common to strains lacking these protein folding factors and hence a similar requirement for DksA.

In summary, this work shows that cytoplasmic PPIs are crucial for the viability of *E*. *coli* under conditions that impact either protein folding or perturb the outer membrane. The viability of strains lacking PPIs requires the presence of DksA and SrrA transcriptional factors, the component of replication/nucleic acid synthesis system Hda/Cmk and MetL. Overexpression of *metL*, *dksA*, *srrA*, *cmk*, *hda, diaA*, chaperone systems encoded by *dnaK*/*J* and *groS*/L and *yjfN* elevates growth defects of Δ6*ppi* bacteria. Importantly, MetL, Cmk and DksA were shown to exhibit the PPIase activity that is inhibited by FK506 and also suppress the conditional lethal phenotype of Δ(*dnaK*/*J tig*) strains. The identification of these three new PPIs provides a sound explanation for the molecular basis of suppression of growth defects of Δ6*ppi* and in part for suppression of Δ(*dnaK*/*J tig*) bacteria. The multicopy suppression by DksA was shown to require adequate levels of GroL/S and RpoE, the intact Tat translocation system and is abolished if either genome integrity or envelope stress responsive pathways are compromised. Furthermore, the coiled-coil domain is not only important for the DksA interaction with RNAP, but also for its PPIase activity as the substitution of conserved residues not only abolished its PPIase activity, but also the ability to exert the multicopy suppression with an acute requirement for D74, F82 and L84 residues of DksA.

## 4. Materials and Methods

### 4.1. Bacterial Strains, Plasmids and Media

The bacterial strains and plasmids used in this study are described in Supplementay Table S3. Luria–Bertani (LB) broth, M9 (Difco, MD, USA) and M9 minimal media were prepared as previously described [17]. When required, media were supplemented with ampicillin (100 μg/mL), kanamycin (50 μg/mL), tetracycline (10 μg/mL), spectinomycin (50 μg/mL) and chloramphenicol (20 μg/mL). For transductions or evaluating growth properties involving either Δ*dksA* derivatives or different cloned *dksA* mutants, M9 minimal medium with or without supplementation of casamino acids was used.

### 4.2. Generation of Null Mutations in Various Genes, Whose Products in the High Dosage Suppress Growth Defects of Δ6ppi Strains and the Construction of Δ(dnaK/J tig) Deletion Derivatives

Non-polar antibiotic-free deletion mutations of various genes, whose overexpression restored the growth of Δ6*ppi* strains, were constructed by using the λ Red recombinase/FLP-mediated recombination system as described previously [14,60]. The antibiotic cassette was amplified using pKD3 and pKD13 as templates [60]. PCR products from such amplification reactions were electroporated into BW25113 containing the λ Red recombinase-encoding plasmid pKD46 (GK1942). Each deletion was verified by PCR amplification and sequencing of PCR products. Such deletions were transduced into parental wild-type strains and Δ6*ppi* strains by bacteriophage P1-mediated transduction. Multiple null combinations were constructed as described previously, followed by the removal of *aph* or *cat* cassettes using the pCP20 plasmid and confirmed to be non-polar [17,60]. When required, additional deletion derivatives were constructed using *ada* cassette for gene replacement using the pCL1920 plasmid as a template in PCR amplification reactions, followed by gene replacement as described above. A non-polar deletion derivative of the *ppk* gene was also constructed in the same manner and verified by its >95% linkage with the *hda* gene. To construct strains simultaneously lacking major chaperones encoded by *dnaK*/*dnaJ* genes and trigger factor PPI encoded by the *tig* gene, previously constructed the deletion strain Δ(*dnaK dnaJ*) GK3078 [14] served as a recipient for the introduction of either *tig*<>*cat* or *tig*<>*ada* deletions by bacteriophage P1-mediated transduction, resulting into the strains SR21830 and SR21836, respectively. Similarly, SR18157 (*dnaK/J*)<>*ada* strain served as a recipient to bring in the *tig*<>*aph* mutation, resulting into the construction of Δ(*dnaK dnaJ tig*) strain SR21842. Transductions were performed on M9 minimal medium at 23 and 30 °C to prevent the accumulation of extragenic suppressors and verified for the inability to plate the bacteriophage λ and also the synthetic lethality on LA medium at 30 °C and above.

### 4.3. The Identification of Multicopy Suppressors, Whose Overexpression Restores the Growth of Either Δ6ppi or Δ(dnaK/J tig) Derivatives under Non-Permissive Growth Conditions

The complete genomic library of all predicted ORFs of *E*. *coli* cloned in pCA24N (ASKA collection) [13] was used to transform the Δ6*ppi* derivative SR18292. As this strain exhibits a stringent growth phenotype on LA medium at all temperatures, LA resistant transformants were selected at 23, 30, 37 and 43 °C in the presence of IPTG (75 μM). DNA insert of all relevant plasmids that restored the growth upon retransformation on LA rich medium at one of these temperatures was sequenced. Since the Δ6*ppi* derivative SR18292 also exhibits the temperature-sensitive phenotype on minimal medium at 43 °C, multicopy suppressors were directly selected for the restoration of growth. In parallel, we used the same genomic library for the isolation of multicopy suppressors that restore the growth on M9 minimal medium with glycerol as the sole carbon source (non-permissive for Δ6*ppi* bacteria) or when supplemented with either 4.5% ethanol or erythromycin (15 μg/mL) or vancomycin (75 μg/mL). Since the ASKA library is a collection of cloned single genes, it is likely that some genes, which are organized as operons and whose products work together, will be missed, the whole genomic plasmid library was constructed after partial digestion of chromosomal DNA of Δ6*ppi* strain by Sau3A I and cloned in a p15-based medium-copy number plasmid as described previously [14].

To isolate multicopy suppressors of Δ(*dnaK/J tig*), deletion derivatives SR21836 and SR21842 were transformed with the above-mentioned genomic library containing all ORFs and plated at 30 and 34 °C on LA medium in the presence of 75 μM IPTG. The plasmid DNA was isolated from cultures obtained from such suppressing clones and used to retransform SR21836 and SR21842 strains to verify the reproducibility of suppression. The identity of gene, which when overexpressed suppresses a Δ(*dnaK/J tig*) derivative, was obtained from DNA sequencing.

### 4.4. The Isolation of Chromosomal Transposon Insertion Mutations That Prevent the Multicopy Suppression by the dksA Gene

As overexpression of the *dksA* gene restores the growth of Δ6*ppi* bacteria, chromosomal gene disruptions were isolated to understand the pathway of DksA-mediated suppression. Thus, the strain SR20561 Δ6*ppi* derivative from SR18292 carrying the *dksA* gene in pBR322 (pSR9332) was used as a host to isolate Tn*10* insertions. In this plasmid, the expression of the full-length *dksA* gene is driven from its own promoters and has an identical insert of the suppressing clone originally isolated as a suppressor of the *dnaKJ* deletion. More than 50,000 transposon insertion mutants were isolated on M9 medium at 37 °C using λTn*10* mutagenesis as described previously [15]. Transductants were screened for a Ts phenotype on LA medium at 42 and 43 °C (lack of suppression). A bacteriophage P1 was grown on such mutants individually and such P1 lysates were used to validate a Ts phenotype in the strain SR20561 (Δ6*ppi* + p*dksA*) by retransduction. Those mutations that bred true were introduced into the wild-type strain BW25113 for further characterization and preparation of chromosomal DNA. The position of Tn*10* was determined by the inverse PCR with nested primers and sequenced using the Tn*10* primer as described previously [14]. Alternatively, the Tn*10* insertion and the flanking chromosomal regions were cloned in a medium-copy vector pMBL18/19 using chromosomal DNA fragments generated by partial digestion by Sau3A I. The position of Tn*10* was determined by sequencing DNA insert using appropriate primers. Temperature sensitive mutations mapping to the *degP* gene were identified either by sequencing the junction of Tn*10* insertion or by P1 transductions using SR8703 (a well characterized *degP*::Tn*10* tet^R^) as a donor. For the analysis of suppression of a Δ*dnaK/J* strain, GK3078 [14] was transformed with the *dksA*-expressing plasmid pSR9332 resulting in SR20733. SR20733 served as a recipient for transductions that were performed on M9 minimal medium at 30 °C. Transductants were subsequently tested for the Ts phenotype at 40 and 42 °C on LA medium.

### 4.5. Cloning of Various Genes for Complementation Studies

For routine complementation, the expression of corresponding genes was induced from clones in the expression vector pCA24N [13]. For the analysis of multicopy suppression by overexpression of the *dksA* gene, the wild type gene was cloned in pBR322 using chromosomal DNA cloned in λ phage from the Kohara library 15A7DNA. Recombinant plasmids carrying the *dksA* gene were selected on the basis of restoration of the growth of either the Δ6*ppi* derivative SR18292 at 43.5 °C on LA medium or that of *dnaKJ* deletion at 42 °C on LA medium. This plasmid pSR9332 is thus identical to the original *dksA* derivative that identified the *dksA* gene [30]. For the complementation of Δ*dnaK/J,* the minimal coding region of the *dnak dnaJ* operon with its native promoter was PCR amplified and cloned in the p15A-based vector, resulting into plasmid pSR21593.

### 4.6. RNA Purification and qRT-PCR Analysis

Exponentially grown cultures of the wild-type strain BW25113, its Δ6*ppi* derivative SR18292, SR20561 (Δ6*ppi* + p*dksA^+^*), GK5165 [(Δ6*ppi* + p*dksA^+^*)::*rpoE*^d^Tn*10*], SR8691 Δ*rpoE* were grown at 37 °C in M9 minimal medium and adjusted to an optical density OD_600_ of 0.05. Culture were allowed to grow up to an OD_600_ of 0.2 and harvested by centrifugation. RNA was purified by the hot phenol extraction procedure as previously described [61]. RNA was precipitated with ethanol and resuspended in 100 μL of DEPC-treated water. RNA amounts were quantified and RNA integrity verified by agarose gel electrophoresis. Quantitative Real Time PCR (qRT-PCR) was used to quantify changes in the *rpoE* gene expression, using the gene-specific primer (Supplementary Table S4). Purified mRNA (2 μg) was converted to cDNA and qRT-PCR was performed using CFX Connect Real-Time PCR Detection System (Bio-Rad, Poland) under conditions described previously [1]. Data were analyzed by software Bio-Rad CFX Maestro. For each experiment, three biological replicates were used.

### 4.7. Protein Purification of Wild-Type and dksA Mutants

For the protein induction, the minimal coding sequence of the *dksA* gene was cloned with an in-frame cleavable N-terminal His_6_ affinity tag in the T7 polymerase-based pET28b expression vector. Specific mutations were introduced by the gene synthesis and Gibson cloning. For the purification, hexa-His-tagged wild-type DksA or its variants cloned in the pET28b expression vector, gene expression was induced in the *E*. *coli* T7 express derivative lacking all six PPIs (SR21984) at 33 °C at an optical density of 0.1 at 600 nm in a 1-l culture by the addition of 0.3 mM IPTG and grown for 4 h. For the purification of Cmk and MetL, the plasmid DNA from JW0893 and JW3911 [13] were used to transform the Δ6*ppi* strain SR18292 to induce the expression of proteins by the addition of 0.3 mM IPTG. After the induction, cells were harvested by centrifugation at 12,000 rpm for 30 min. The pellet was resuspended in B-PER reagent (Pierce) and adjusted to contain 50 mM NaH_2_PO_4_, 300 mM NaCl, 10 mM imidazole (buffer A), supplemented with lysozyme to a final concentration of 200 μg/mL, a cocktail of protease inhibitors (Sigma, Poznan, Poland) and 30 units of benzonase (Merck, Poznan, Poland). The mixture was incubated on ice for 45 min with gentle mixing. The lysate was centrifuged at 45,000× *g* for 30 min at 4 °C. Soluble proteins were applied over nickel-nitrilotriacetic acid beads (Qiagen, Geneva, Switzerland), washed and eluted with buffer A with a linear gradient (50 mM–500 mM) of imidazole.

### 4.8. The PPIase Assay and the PPIase-Dependent Refolding of RNase T1

For the measurement of PPIase activity with purified individual wild-type or mutant protein, PPIs were used at a concentration in the range of 0.1 to 5 μM. All purified proteins were obtained from Δ6*ppi* strains. The PPIase activity was measured in a chymotrypsin-coupled enzymatic assay [23,36] using 8 mM *N*-Suc-Ala-Ala-*cis*-Pro-Phe-*p*-nitroanilide as the substrate. The PPIase activity was determined in 35 mM HEPES pH 8.0 as assay buffer and the activity was measured at 10 °C. The reaction was initiated by the addition of chymotrypsin (300 μg/mL) and change in the absorbance at 390 nm was recorded using Specord 200 Plus spectrophotometer equipped with the Peltier temperature control system. When required, the FK506 inhibitor (Sigma, Poznan, Poland) was added in a 2-fold molar excess. FK506 and purified DksA, MetL and Cmk were incubated at 10 °C for 5 min prior to the addition of chymotrypsin.

The proline-limited folding of ribonuclease T1 (RNase T1) was analyzed using the procedure [23] with few modifications. The purified RNase T1 was purchased from Worthington (USA). To unfold RNase, T1 was incubated at 64 μM concentration in a buffer containing 100 mM Tris-HCl, pH 8.0 and 8 M urea for 2 h at 25 °C. The refolding was initiated by 40-fold dilution of urea in a buffer containing 100 mM Tris-HCl, pH 8.0 with the final concentration of 0.33 mM RNase T1. The folding catalyst was added to the reaction mixture at a 10 μM concentration prior to the urea dilution. The tryptophan fluorescence was measured with the emission wavelength of 320 nm (20-nm bandwidth) and the excitation wavelength of 268 nm (20-nm bandwidth) using a Tecan Spark 10M spectrofluorophotometer. The measurement was performed for 15 min with interval time of 1 s.

### 4.9. β-Galactosidase Activity Assay and Measurement of GroL Levels

For the quantification of activity of the *rrnB*P1 promoter, a single-copy promoter fusion was constructed. To achieve this, the minimal *rrnB*P1 promoter region was cloned in the promoter probe vector pRS415 and transferred to the chromosome using bacteriophage λ derivative λRS45 to generate a single copy *rrnB*P1-*lacZ* fusion in BW25113, using previously described procedure [61,62]. This resulted in the construction of SR22123 strain, which subsequently served as a host to introduce the null allele of the *dksA* gene (SR22415) and various plasmids carrying either the wild-type *dksA* gene or its different mutant derivatives. For the measurement of *β*-galactosidase activity, isogenic cultures were grown in LB medium at 37 °C. Exponentially grown cultures were adjusted to an OD_600_ of 0.05 and aliquots of samples were used to measure the *β*-galactosidase activity as described [15]. For the measurement of GroL levels, overnight cultures of the wild-type strain and its isogenic derivatives carrying either *groSp*^d^*1* or *groSp*^d^*2* mutation were grown at 30 °C in M9 minimal medium. Cultures were adjusted to an OD_600_ of 0.05 in M9 medium and allowed to reach an OD_600_ of 0.2. One-milliliter aliquots were shifted to prewarmed tubes held at 42 °C and incubated for 15 min. Proteins were precipitated by TCA (10%) and analyzed on 12.5% SDS-PAGE, followed by immunoblotting with anti-GroL antibodies.

## Figures and Tables

**Table 1:** The sensitivity of a Δ6*ppi* derivative on M9 medium supplemented with different agents at 37 °C. Exponentially grown cultures were adjusted to an optical density OD_600_ of 0.2 and spot diluted. Numbers indicate the colony forming units of a Δ6*ppi* derivative as compared to the wild type.

Strain	Ethanol 4%	SDS 1%	Deoxycholate 0.75%	Vancomycin 60 μg/mL	Erythromycin 12 μg/mL	Tetracycline 1.5 μg/mL	Kanamycin 2 μg/mL
wild type	8 × 10^8^	3 × 10^8^	6 × 10^8^	2 × 10^8^	6 × 10^8^	3 × 10^8^	7 × 10^8^
Δ6*ppi*	2 × 10^2^	3 × 10^2^	-	-	-	3 × 10^2^	1 × 10^2^

**Table 2:** Multicopy suppressors that restore the growth of Δ6*ppi* strains under different conditions.

	Growth conditions							
Gene	LA 23 °C	LA 30 °C	LA 37 °C	LA 42 °C	LA 43.5 °C	V/Et/Er	M9 42 °C	Function
*metL*	+ ^a^	+	+	+	+	+ V/Er	+ sc	aspartokinase
*dksA*	-	-	± ^b^	+	+	- ^c^	+	transcription
*srrA*	-	+ sc	+	+	+	+ Et	+	transcription
*yjfN*	-	+ sc	+	+	+	+ Er	+	proteolytic
*cmk*	+	+	+	+	±	+ Et/Er/V	+	DNA synthesis
*hda*	+	+	+ sc	-	-	+ Et/Er	+	replication
*diaA*	+	+	+	-	-	+ Et/Er	+	replication
*dnaK/J*	+	+	+	+	±	NT	-	chaperone
*groL/S*	-	± sc	+	+	±	NT	+	chaperonin
*mqsA*	-	+	+	+	±	+ Er	+	antitoxin
*yceD*	-	±	+	-	-	+ V	±	stress response
*ariR*	+	+	+	+	+	+ Er	+ sc	stress response
*pepA*	+	+	+	+	+ sc	-	±	proteolytic
*bcr*	+	+	+	-	-	+ Et/Er	-	peptide transport
*cspC*	-	±	+	+ sc	+ sc	-	+	stress response
*hha*	-	-	-	-	-	+ Er	+	transcription with *mqs*
*mppA*	+	+	± sc	-	-	+ V/Er	-	murein binding
*ycaD*	+	+	+ sc	-	-	+ V/Er	-	transport
*murI*	+ sc	±	+	-	-	+ Et/Er/V	-	peptidoglycan
*nudE*	-	+	+	-	-	+ Et/Er	-	nudix
*nudJ*	+	+	±	-	-	-	-	nudix
*yigB*	+	+	-	-	-	-	-	riboflavin
*ycjW*	-	-	+	-	-	+ Er	-	transcription
*gadW*	± sc	±	± sc	-	-	+ Er	-	acid resistance
*ydgC*	+	+	+	±	-	+ Er	-	GlpM family
*yhaM*	-	-	+	-	-	-	-	cysteine detoxification
*preT*	-	-	+	-	-	-	-	pyrimidine metabolism

Additional suppressors, which restored the growth by 10-fold on LA at 37 °C (±), include *hchA*, *hslO*, *ilvB*, *rluB*, *cspH*, *argA*, *msyB, ddpF* and *intQ*. However, the *msyB* gene was also isolated as a multicopy suppressor for the growth on M9 minimal medium with glycerol as the sole carbon source. +^a^ indicates the wild type-like growth efficiency of plating close to 1. ±^b^ indicates up to 50% growth as compared to the wild type. -^c^ indicates the inability to support the colony forming capacity. Note overexpression of *dnaK/J* and *groS/L* genes restores the growth at 42 °C, but not above 43 °C. sc, small colonies; NT, not tested; V, vancomycin (75 μg/mL); Et, ethanol (4.5%); Er, erythromycin (15 μg/mL).

**Table 3:** The essentiality of *dksA*, *srrA*, *hda*, *cmk*, *metL* genes and a partial requirement for HchA in Δ6*ppi* strains.

Number of Transductants in Δ6*ppi* Strains Obtained Either in the Presence or Absence of Covering Wild-Type Plasmid-Born Gene		
Gene	BW25113 LA/M9 + CAA 37 °C	MC4100 LA/M9 + CAA 37 °C
Δ*hda*	9	6
Δ*hda* +p*hda*^+^	1234	1146
Δ*dksA*	8 *	small non-viable colonies
Δ*dksA+*p*dksA*^+^	1436	1598
*srrA*	7	455 small colonies after 48 h, cold sensitive at 23 °C and 30 °C
*srrA+*p*srrA*^+^	1255	1376
*metL*	11 *	23
*metL*+p*metL*^+^	1830	1941
*cmk*	136 small colonies after 48 h	254 small colonies after 48 h
*cmk+*p*cmk*^+^	1575	945
*hchA*	312 small colony size	380 after 48 h (however, viable on M9)
*hchA+*p*hchA*^+^	1174	1470

* Additional non-viable transductants obtained when either Δ*dksA* or Δ*metL* were introduced, which do not grow upon streaking. Note viable very small colony-sized transductants were obtained at the normal frequency, when Δ*ariR*, Δ*cspC*, Δ*pepA* or Δ*ycjW* were introduced in the Δ6*ppi* background after 24 h incubation. The comparable transductional frequency with or without covering the plasmid obtained when either Δ*diaA* or Δ*yjfN* were introduced. CAA indicates supplementation of minimal medium by 0.2% casamino acids.

**Table 4:** The essentiality of the *dksA gene* in Δ6*ppi* as determined by the linked *htrE* mutation.

Number of Transductants with Selection for Tetracycline Resistance		
Donor	Recipient	
	**BW25113**	Δ6*ppi*
*htrE*::tet	873 tet^R^	912 tet^R^
*htrE*::tet *dksA*::cm	940 tet^R^	922 tet^R^
	(870 cm^R^)	(0 cm^R^)

**Table 5:** Properties of various DksA mutants in terms of suppression of auxotrophic phenotype of a Δ*dksA* strain, the suppression of Ts phenotype and impact on the PPIase activity.

Strain	Auxotrophy	*rrnB*P1 Activity	Δ*dnaK/J*	Δ(*dnaK/J tig*)	Δ6*ppi*	PPIase Activity
wild type DksA	+^a^	repressed	+	+	+	+
DksA D74N	-^b^	not repressed	-^e^	-^e^	-	reduced
DksA F82Y	± ^f^ sc	repressed	-^e^	-^e^	-	highly reduced
DksA S83A	-^c^	weakly repressed	-^e^	-^g^	-	reduced
DksA L84A	-^b^	not repressed	-^e^	-^e^	-	reduced
DksA E85A	-^c^	weakly repressed	-	-^g^	-^d^	marginal reduction

+^a^ indicates the complementation of auxotrophic growth on minimal medium of Δ*dksA* bacteria. -^b^ indicates no growth at all on minimal medium, the tight auxotrophic phenotype. -^c^ indicates the background growth, but no single colonies on minimal medium. -^d^ indicates a leaky small colony phenotype at 43.5 °C after the prolonged incubation of transformants of Δ6*ppi.* -^e^ indicates no suppression of Ts phenotype of Δ*dnaK/J* mutant bacteria at 42 °C. -^f^ indicates small colonies after 48 h incubation and sc indicates small colonies. -^g^ indicates small colonies after 48 h incubation of Δ(*dnaK/J tig*) bacteria at 30 °C.

**Table 6:** Tn*10* insertions that block the multicopy suppression by DksA of Δ6*ppi*, their suppression of Δ*dnaK/J* strains as determined by colony forming units, the synthetic phenotype in the Δ*dksA* background and the lack of *ppk* requirement for the DksA-mediated suppression of Δ6*ppi*.

Gene	Tn*10* Position	Δ*dnaK/J*+p*dksA*^+^		wt	Δ*6ppi*	Δ*dksA*	Function
		40 °C	42 °C	43 °C	M9 37 °C	43 °C	
*rpoEp* ^d^	−90	-	-	+^a^ sc	+	+ sc	sigma factor
*groSp* ^d^ *1*	−101	-	-	-	+ sc	-	chaperone
*groSp* ^d^ *2*	−101	-	-	+ sc	+	+	chaperone
*degP*	12/112 *	-	-	-	+	- Ts >39 °C	periplasmic protease
*lepA*	702	± sc	-	±^b^ sc	+ sc	-^c^	translational GTPase
*ahpC*	94	-	-	+	+ sc	+	oxidative stress
*rnhA*	59	-	-	+	+ sc	± sc	ribonuclease HI
*tatC*	23	-	-	+	+	- sc	transport of folded proteins
*tolA*	299	± sc	-	+	± sc	± vsc	cell envelope integrity
*mrcB*	1186	± sc	-	+	+	+	peptidoglycan synthesis
*oxyR*	518	± sc	-	+	+	+	oxidative stress regulator
*cpxR*	474	+	± sc	+	+	+ sc	envelope stress regulator
*cydA*	20	-	-	-	+	-	cytochrome *d* terminal oxidase
*clsA*	533	± sc	-	+	+	+	cardiolipin synthase
*ftsX*	166	-	-	± sc	± sc	-	cell division
*nudB*	369	+	±	+	+	± sc	folate biosynthesis
*envZ*	291	± sc	-	+	+	+	regulation of *ompF*/*C* expression
*phoU*	655	-	-	+sc	+sc	- vsc	P_i_ signalling
Δ*ppk*	deletion	+	-	+	+	+	polyphosphate kinase

+^a^ indicates the normal growth. ±^b^ indicates the partial temperature sensitivity. -^c^ indicates the inability to support the colony-forming ability. * indicates the Tn*10* insertion position in the *degP* gene determined for two out of nine mutants. sc, small colonies; vsc, very small colonies after the prolonged incubation.

## Supplementary Materials

Supplementary materials can be found at https://www.mdpi.com/1422-0067/21/16/5843/s1. Table S1. Overexpression of *groS*/*L* genes can rescue growth of Δ6*ppi* bacteria up to 42 °C on LA medium; Table S2. Growth rate per hour (μ) of strains carrying Tn*10* insertions in various genes that prevent the DksA-mediated suppression to different extents in Δ6*ppi* and Δ*dnaK/J* derivatives carrying the plasmid expressing the *dksA* gene; Table S3. Bacterial strains and plasmids used in this study; Table S4. Primers; Figure S1. Overexpression of the *nudJ* and *ydgC* genes can restore the wild type-like growth of Δ6*ppi* bacteria on LA medium at either 23 or 30 °C.

## Abbreviations

PPI	peptidyl-prolyl *cis*/*trans* isomerase
FKBP	FK506-binding protein
RNAP	RNA polymerase
