# Supplementary material for: Multicopy Suppressor Analysis of Strains Lacking Cytoplasmic Peptidyl-Prolyl cis/trans Isomerases Identifies Three New PPIase Activities in Escherichia coli That Includes the DksA Transcription Factor
Source: Int J Mol Sci. 2020 Aug 14;21(16):5843. doi: 10.3390/ijms21165843 (PMC7461557; doi:10.3390/ijms21165843)
Supplement: Supplementary file 1 [file ijms-21-05843-s001.pdf]

Multicopy suppressor analysis of strains lacking cytoplasmic peptidyl-prolyl *cis/trans* isomerases identifies three new PPIase activities in *Escherichia coli* that includes the DksA transcription factor

Pawel Wojtkiewicz, Daria Biernacka, Patrycja Gorzelak, Anna Stupak, Gracjana Klein*, and Satish Raina*

Unit of Bacterial Genetics, Gdansk University of Technology, Narutowicza 11/12, 80-233 Gdansk, Poland
* Correspondence: gracjana.klein@pg.edu.pl (G.K.); satish.raina@pg.edu.pl (S.R.);
Tel.: +48-58-347-2618 (G.K. & S.R.)

Keywords: prolyl isomerase; protein folding; RNA polymerase; DksA; heat shock proteins; DnaK/J; GroL/S; RpoE sigma factor; RNase H; LepA

Supplementary Materials

Table S1. Overexpression of *groS/L* genes can rescue growth of Δ6*ppi* bacteria up to 42 °C on LA medium. The numbers indicate the colony forming ability at different temperatures as determined by spot-dilution assay.

strain	37 °C	42 °C	43.5 °C
Δ6*ppi*+vector	10^2	$1.1x10^1$	-
Δ6*ppi*+p*groS/L*$^+$	$3x10^7$	$2x10^7$	$3x10^3$
Δ6*ppi*+p*dnaK/J*$^+$	$7x10^7$	$2x10^7$	$4x10^3$
Δ6*ppi*+p*dksA*$^+$	$4x10^3$	$6x10^7$	$5x10^7$

Table S2. Growth rate per hour (μ) of strains carrying Tn*10* insertions in various genes that prevent the DksA-mediated suppression to different extents in Δ6*ppi* and Δ*dnaK/J* derivatives carrying the plasmid expressing the *dksA* gene.

strain	Growth rate per hour (μ)
Δ6*ppi*+p*dksA*$^+$	0.60
Δ6*ppi*+p*dksA*$^+$ *ahpC*::Tn*10*	0.44
Δ6*ppi*+p*dksA*$^+$ *ppk*::Cm	0.51
Δ6*ppi*+p*dksA*$^+$ *cpxR*::Tn*10*	0.53
Δ6*ppi*+p*dksA*$^+$ *nudB*::Tn*10*	0.37
Δ6*ppi*+p*dksA*$^+$ *lepA*::Tn*10*	0.32
Δ6*ppi*+p*dksA*$^+$ *tatC*::Tn*10*	0.20
Δ6*ppi*+p*dksA*$^+$ *phoU*::Tn*10*	0.51
Δ6*ppi*+p*dksA*$^+$ *groSp*^{d}2::Tn*10*	0.41
Δ6*ppi*+p*dksA*$^+$ *rnhA*::Tn*10*	0.23
Δ6*ppi*+p*dksA*$^+$ *envZ*::Tn*10*	NL[a]
Δ6*ppi*+p*dksA*$^+$ *rpoEp*^{d}::Tn*10*	NL
Δ*dnaKJ*+p*dksA*$^+$	0.45
Δ*dnaKJ*+p*dksA*$^+$ *ppk*::Cm	0.38
Δ*dnaKJ*+p*dksA*$^+$ *envZ*::Tn*10*	0.58
Δ*dnaKJ*+p*dksA*$^+$ *lepA*::Tn*10*	0.33
Δ*dnaKJ*+p*dksA*$^+$ *tolA*::Tn*10*	0.31
Δ*dnaKJ*+p*dksA*$^+$ *tatC*::Tn*10*	0.22
Δ*dnaKJ*+p*dksA*$^+$ *groSp*^{d}2::Tn*10*	NL
Δ*dnaKJ*+p*dksA*$^+$ *phoU*::Tn*10*	NL
Δ*dnaKJ*+p*dksA*$^+$ *rnhA*::Tn*10*	NL
Δ*dnaKJ*+p*dksA*$^+$ *rpoEp*^{d}::Tn*10*	NL

NL[a] indicates non-linear growth and hence data not used to calculate growth rate.

Table S3. Bacterial strains and plasmids used in this study

Strains/Plasmids	Relevant characteristic	Reference or source
**Strains**		
BW25113	$lacI^{q}$ $rrnB_{T14}$ $\Delta lacZ_{WJ16}$ *hsdR514* $\Delta araBAD_{AH33}$ $\Delta rhaBAD_{LD78}$	[60]
MC4100	F^{-} *araD139* Δ(*argF-lac*)*U169*	Our collection
T7 Express *lysY/I*^{q}	MiniF *lysY fhuA2 lacZ::T7 gene1 [lon] ompT*	NEB
GK1942	BW25113 (pKD46)	[14]
SR18157	BW25113 (*dnaK dnaJ*)<>*ada*	This study
SR18292	SR18272 *fkpB*<>*aph* (Δ*6ppi*)	[1]
GK4034	SR18292 *fkpB*<>*frt* (Δ*6ppi*)	[1]
SR18255	MC4100 *ppiB*<>*frt ppiC*<>*frt tig*<>*frt slyD*<>*frt fklB*<>*frt fkpB*<>*frt* (Δ*6ppi*)	This study
GK4649	MC4100 *ppiB*<>*frt ppiC*<>*frt tig*<>*frt slyD*<>*frt fklB*<>*frt fkpB*<>*frt* (Δ*6ppi*)	This study
SR21984	T7 Express *lysY/I*^{q} *ppiB*<>*frt ppiC*<>*frt tig*<>*frt slyD*<>*frt fklB*<>*frt fkpB*<>*frt* (Δ*6ppi*)	[1]
SR20561	SR18292 + pSR9332 (p*dksA*$^{+}$)	This study
SR20900	SR20561 *rpoE*::Tn*10* (*rpoEp*^{d}1)	This study
GK5165	SR20561 *rpoE*::Tn*10* (*rpoEp*^{d}2)	This study
GK5109	SR20561 *groS*::Tn*10* (*groSp*^{d}1)	This study
GK5578	SR20561 *groS*::Tn*10* (*groSp*^{d}2)	This study
GK5084	SR20561 *clsA*::Tn*10*	This study
GK5087	SR20561 *ahpC*::Tn*10*	This study
GK5110	SR20561 *nudB*::Tn*10*	This study
GK5147	SR20561 *lepA*::Tn*10*	This study
GK5162	SR20561 *envZ*::Tn*10*	This study
GK5166	SR20561 *ftsX*::Tn*10*	This study
GK5182	SR20561 *degP*::Tn*10*	This study
GK5183	SR20561 *rnhA*:: Tn*10*	This study
GK5193	SR20561 *cydA*::Tn*10*	This study
GK5196	SR20561 *oxyR*::Tn*10*	This study
GK5198	SR20561 *mrcB*::Tn*10*	This study
GK5374	SR20561 *pstS*::Tn*10*	This study
GK5577	SR20561 *phoU*::Tn*10*	This study
GK5621	SR20561 *cpxR*::Tn*10*	This study
GK5688	SR20561 *tolA*::Tn*10*	This study
GK5690	SR20561 *tatC*::Tn*10*	This study
GK5347	BW25113 *rpoE*::Tn*10* (*rpoEp*^{d})	This study
SR8691	BW25113 *rpoE*<>*cat*	This study
SR8703	BW25113 *degP*::Tn*10*	Our collection
GK3078	BW25113 (*dnaK dnaJ*)::kan	[14]
SR20733	GK3078 + pSR9322 (p*dksA*$^{+}$)	This study
SR21830	GK3078 *tig*<>*cat*	This study
SR21836	GK3078 *tig*<>*ada*	This study
SR18157	BW25113 (*dnaK dnaJ*)<>*ada*	This study
SR19522	BW25113 *tig*<>*aph*	This study
SR21842	SR18157 *tig*<>*aph*	This study
GK2549	BW25113 *degP*<>*ada*	[14]
SR20347	BW25113 *srrA*<>*cat*	This study
SR20360	BW25113 *cmk*<>*cat*	This study
SR20364	BW25113 *hda*<>*cat*	This study
SR21070	BW25113 *yjfN*<>*cat*	This study
SR21640	BW25113 *hchA*<>*cat*	This study

GK5866	BW25113 *dksA*<>*cat*	This study
SR20355	GK5866 *htrE*::tet	This study
SR532	CA8000 *htrE*::tet	[18]
SR20066	BW25113 *dksA*<>*aph*	This study
GK5654	SR20066 *rnhA*::Tn*10*	This study
GK5666	SR20066 *lepA*::Tn*10*	This study
GK5680	SR20066 *degP*::Tn*10*	This study
GK5692	SR20066 *cydA*::Tn*10*	This study
SR22019	BW25113 *metL*<>*aph*	This study
SR22252	BW25113 *ppk*<>*cat*	This study
SR22265	SR18292 *ppk*<>*cat*	This study
SR22300	SR20733 *ppk*<>*cat*	This study
SR22407	SR20561 *ppk*<>*cat*	This study
SR22123	BW25113 *rrnB*P1-*lacZ*	This study
SR22198	SR22123 *dksA*<>*aph*	This study
SR22415	SR22198 *dksA*<>*frt*	This study
SR22423	SR22415+p*dksA*$^+$	This study
SR22425	SR22415+p*dksA* D74N	This study
SR22427	SR22415+p*dksA* F82Y	This study
SR22430	SR22415+p*dksA* S83A	This study
SR22431	SR22415+p*dksA* L84A	This study
SR22433	SR22415+p*dksA* S83A	This study
**Plasmids**		
pET28b	expression vector	Our collection
pMBL18	expression vector	Our collection
pOK12	expression vector	Our collection
pBR322	cloning vector	Our collection
pCP20	ts replicon with inducible FLP recombinase	[60]
pKD3	*oriR6K*$_g$, *bla*(Amp^R), *kan*, *rgnB*(Ter), *cat*	[60]
pKD13	*oriR6K*$_g$, *bla*(Amp^R), *kan*, *rgnB*(Ter)	[60]
pKD46	*araBp-gam-bet-exo*, *bla*(Amp^R), *repA101*(ts)	[60]
pCA24N	IPTG-inducible expression vector cm^R	[13]
pRS415	*lacZYA* transcriptional fusion vector	[62]
pSR21593	pMBL18 *dnaK/J*	This study
pSR9147	pMBL18 *groL/S*	This study
pSR9332	*dksA*$^+$ in pBR322 amp^R	This study
pSR22206	*dksA*$^+$ in pET28b kan^R	This study
pSR22212	*dksA* D74N in pET28b kan^R	This study
pSR22215	*dksA* F82Y in pET28b kan^R	This study
pSR22218	*dksA* S83A in pET28b kan^R	This study
pSR22224	*dksA* L84A in pET28b kan^R	This study
pSR22230	*dksA* E85A in pET28b kan^R	This study
pSR22189	*dksA*$^+$ in pBR322 *lacI*^{q} tet^S amp^R	This study
pSR22505	*dksA* D74N in pBR322 *lacI*^{q} tet^S amp^R	This study
pSR22511	*dksA* F82Y in pBR322 *lacI*^{q} tet^S amp^R	This study
pSR22519	*dksA* S83A in pBR322 *lacI*^{q} tet^S amp^R	This study
pSR22498	*dksA* L84A in pBR322 *lacI*^{q} tet^S amp^R	This study
pSR22525	*dksA* E85A in pBR322 *lacI*^{q} tet^S amp^R	This study
JW0141	*dksA*$^+$ in pCA24N cm^R	[13]
JW3911	*metL*$^+$ in pCA24N cm^R	[13]
JW0893	*cmk*$^+$ in pCA24N cm^R	[13]
pSR21129	*srrA*$^+$ in pBR322 amp^R	This study

Table S4. Primers

For gene disruption:

dnaKJ spec For	5′-CAG ACT CAC AAC CAC ATG ATG ACC GAA TAT ATA GTG GAG ACG TTT AGA TGG CAC CTT GCC GTA GAA GAA C-3′
dnaKJ spec Rev	5′-GCC TGC CCA CGG GCA GGC TTT TGG GGA GGT TAG CGG GTC AGG TCG TCA AAT AAC GCT TGA GTT AAG CCG C-3′
tig spec For	5′-TGC GGG GTA AGA GTT GAC CGA GCA CTG TGA TTT TTT GAG GTA ACA AGA TGG CAC CTT GCC GTA GAA GAA C-3′
tig spec Rev	5′-TGC GAA TTT AGC GCG TTA TGC TGC GTA AAT TAC GCC TGC TGG TTC ATC AGT AAC GCT TGA GTT AAG CCG C-3′
tig cm For	5′-TGC GGG GTA AGA GTT GAC CGA GCA CTG TGA TTT TTT GAG GTA ACA AGA TGC ATA TGA ATA TCC TCC TTA G-3′
tig cm Rev	5′-TGC GAA TTT AGC GCG TTA TGC TGC GTA AAT TAC GCC TGC TGG TTC ATC AGG TGT AGG CTG GAG CTG CTT C-3′
diaA spec For	5′-TAA GGA TGC CTT TAA TGA CCA CTC ATA ATT AAG GTT TAA GGA TTA GCG TGG CAC CTT GCC GTA GAA GAA C-3′
diaA spec Rev	5′-ATT GGC GAT AAT GCC TTC ATG TAT TCT CCT TAA TCA TCC TGG TGA GGG AAT AAC GCT TGA GTT AAG CCG C-3′
srrA cm For	5′-GCC CTG AAA AGT TAA CGA CAG GCT CCT GAA AAG GAG TGT TTT TTT TCA TGC ATA TGA ATA TCC TCC TTA G-3′
srrA cm Rev	5′-TGG CCC GGT CAC CAC GAT GGC AAA ACG CAT TAC TTA TCT TGC CCC TGG AAG TGT AGG CTG GAG CTG CTT C-3′
cmk cm For	5′-AAT GCG CGC GGT TAT GTT AAC GGT ACG CCT GTT TTA AGG AGA TAA AGA TGC ATA TGA ATA TCC TCC TTA G-3′
cmk cm Rev	5′-TGC AAC GGG GGT ACT GCA AAT TCG GTC GCT TAT GCG AGA GCC AAT TTC TGG TGT AGG CTG GAG CTG CTT C-3′
dksA cm For	5′-TTT CCC CCG AAC ATG GGG ATC GAT AGT GCG TGT TAA GGA GAA GCA ACA TGC ATA TGA ATA TCC TCC TTA G-3′
dksA cm Rev	5′-CTG TGG TAA ACG TGA TGG AAC GGC TGT AAT TAG CCA GCC ATC TGT TTT TCG TGT AGG CTG GAG CTG CTT C-3′
hda cm For	5′-CTG TGG TAA ACT TCT CGC GAT TTT GTG AAA TCC TGG TTG AGG TAT CTC TGC ATA TGA ATA TCC TCC TTA G-3′
hda cm Rev	5′-CGC GCC GCA TCC GAC AAT AAA CAC CTT ATC TAC AAC TTC AGA ATT TCT TTG TGT AGG CTG GAG CTG CTT C-3′
yjfN cm For	5′-GAT GGA ATG CCC TGC AAC ATG TGG GGA AAC GAA AAA TGG AGC TGA CGA TGC ATA TGA ATA TCC TCC TTA G-3′
yjfN cm Rev	5′-CGG CAT TGT TCA TTT GCT GTA GAT GAC ACT CAT GCA TAC AGT ATC GCC TGG TGT AGG CTG GAG CTG CTT C-3′
hchA cm For	5′-CGC AAA TAT AGT GAC TAC CCT AAC TAA GCA ACA ATA AGG AAT ACA CTA TGC ATA TGA ATA TCC TCC TTA G-3′
hchA cm Rev	5′-TAT GCG CTT ACA TTC AAA CGT AAC AGG GAT TAA CCC GCG TAA GCT GCC AGG TGT AGG CTG GAG CTG CTT C-3′

ariR cm For	5′-AAT AAA CAG AAT ACA TTA AAA TTT CAT AAG TAA GAT GAG AGG T TA CCA TGC ATA TGA ATA TCC TCC TTA G-3′
ariR cm Rev	5′-TCT ATA AAC TTA TAC TTA ATA ATT AGA AGT TAC ATA TCA TCA GCT GTG TAG TGT AGG CTG GAG CTG CTT C-3′
yhaM cm For	5′-CGT TCC TGG CAT TTT CTT GAT TTA CCT GAA ATT TTA AGG TTT TTA ATA TGC ATA TGA ATA TCC TCC TTA G-3′
yhaM cm Rev	5′-CAT CCG GCA CGA TCC CCA AAA CCT GGC GTT TAT CTG GCC TTG CTC GCC ATG TGT AGG CTG GAG CTG CTT C-3′
ppk cm For	5′-ATA ATA TCC AGG CAG TGT CCC GTG AAT AAA ACG GAG TAA AAG TGG TAA TGC ATA TGA ATA TCC TCC TTA G-3′
ppk cm Rev	5′-GAG GGG ATT TAT CGT GTA TTG GCA TAG GGT TAT TCA GGT TGT TCG AGT GAG TGT AGG CTG GAG CTG CTT C-3′
metL cm For	5′-ATT GCC GAC CTG GAA AAT GGC TTC CGG GCT GCA AAC AAG GGG TAA AAA TGC ATA TGA ATA TCC TCC TTA G-3′
metL cm Rev	5′-GAG TAC CGG GCA TTA TTA AAT TTC TGA AAT TAC AAC AAC TGT GCC AGC CGG TGT AGG CTG GAG CTG CTT C-3′

For cloning and overexpression of specific genes:

dnaKJ For	5′-GCC TTG GAT CCG ATT CAT TCT TTA TAT G-3′
dnaKJ Rev	5′-CAT ATC TTC AAC GGA TCC TAT TTT TAC C-3′
dksA For	5′-AGG AGA AGC ACC ATG GAA GAA GGG CA-3′
dksA Rev	5′-GAT GGA ACG GCT GCT CGA GGC CAG CCA TCT G-3′

For q-RT-PCR:

q*rpoE* For	5′-TGG CTG TAT CGG ATT GCT GTA A-3′
q*rpoE* Rev	5′-AGT TTT CAG CTT CAA TGG CAT CC-3′

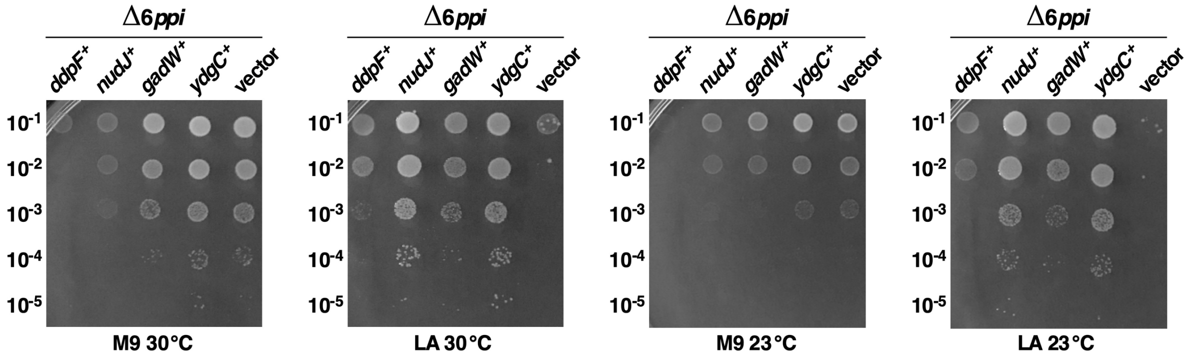

Figure S1. Overexpression of the *nudJ* and *ydgC* genes can restore the wild type-like growth of Δ6*ppi* bacteria on LA medium at either 23 or 30 °C. Isogenic cultures of Δ6*ppi* bacteria carrying either the vector alone or the plasmid encoding different multicopy suppressing genes were adjusted to an OD_{600} of 0.1 and serially spot diluted on M9 minimal medium and LA medium in the presence of 75 μM IPTG at indicated temperatures. Plates were incubated for 48 h and data from a representative experiment are presented.
